# Fibrogenic Activity of MECP2 Is Regulated by Phosphorylation in Hepatic Stellate Cells

**DOI:** 10.1053/j.gastro.2019.07.029

**Published:** 2019-11

**Authors:** Eva Moran-Salvador, Marina Garcia-Macia, Ashwin Sivaharan, Laura Sabater, Marco Y.W. Zaki, Fiona Oakley, Amber Knox, Agata Page, Saimir Luli, Jelena Mann, Derek A. Mann

**Affiliations:** Newcastle Fibrosis Research Group, Institute of Cellular Medicine, Faculty of Medical Sciences, Newcastle University, Newcastle upon Tyne, United Kingdom

**Keywords:** MCM, lncRNA, Myofibroblast, Epigenetic Factor, APAP, *N*-acetyl-ρ-aminophen, BrdU, bromodeoxyuridine, CCl_4_, carbon tetrachloride, ECM, extracellular matrix, HSC, hepatic stellate cells, IL, interleukin, IPA, Ingenuity Pathway Analysis, KEGG, Kyoto Encyclopedia of Genes and Genomes, lncRNA, long noncoding RNA, MCM, maintenance protein complex, MMP, matrix metalloproteinase, mRNA, messenger RNA, MTT, 3-(4,5-dimethylthiazol-2-yl)-2,5-diphenyltetrazolium bromide, PCA, principal components analysis, qRT-PCR, quantitative reverse-transcription polymerase chain reaction, SEM, standard error of the mean, α-SMA, α–smooth muscle actin, siRNA, small interfering RNA, TGF, transforming growth factor, WT, wild type

## Abstract

**Background & Aims:**

Methyl-CpG binding protein 2, MECP2, which binds to methylated regions of DNA to regulate transcription, is expressed by hepatic stellate cells (HSCs) and is required for development of liver fibrosis in mice. We investigated the effects of MECP2 deletion from HSCs on their transcriptome and of phosphorylation of MECP2 on HSC phenotype and liver fibrosis.

**Methods:**

We isolated HSCs from *Mecp2*^*–/y*^ mice and wild-type (control) mice. HSCs were activated in culture and used in array analyses of messenger RNAs and long noncoding RNAs. Kyoto Encyclopedia of Genes and Genomes pathway analyses identified pathways regulated by MECP2. We studied mice that expressed a mutated form of *Mecp2* that encodes the S80A substitution, MECP2S80, causing loss of MECP2 phosphorylation at serine 80. Liver fibrosis was induced in these mice by administration of carbon tetrachloride, and liver tissues and HSCs were collected and analyzed.

**Results:**

MECP2 deletion altered expression of 284 messenger RNAs and 244 long noncoding RNAs, including those that regulate DNA replication; are members of the minichromosome maintenance protein complex family; or encode CDC7, HAS2, DNA2 (a DNA helicase), or RPA2 (a protein that binds single-stranded DNA). We found that MECP2 regulates the DNA repair Fanconi anemia pathway in HSCs. Phosphorylation of MECP2S80 and its putative kinase, HIPK2, were induced during transdifferentiation of HSCs. HSCs from MECP2S80 mice had reduced proliferation, and livers from these mice had reduced fibrosis after carbon tetrachloride administration.

**Conclusions:**

In studies of mice with disruption of *Mecp2* or that expressed a form of MECP2 that is not phosphorylated at S80, we found phosphorylation of MECP2 to be required for HSC proliferation and induction of fibrosis. In HSCs, MECP2 regulates expression of genes required for DNA replication and repair. Strategies to inhibit MECP2 phosphorylation at S80 might be developed for treatment of liver fibrosis.

**What You Need to Know**

**Background and context**

During development of liver fibrosis, hepatic stellate cells differentiate into myofibroblasts. This process is regulated by epigenetic events, including those controlled by the methyl-CpG binding protein 2 (MECP2).

**New findings**

MECP2 regulates expression of mRNAs and long noncoding RNAs in hepatic myofibroblasts, including those that control DNA replication. MECP2 is phosphorylated at S80; mutation of this amino acid protects mice from toxin-induced liver fibrosis.

**Limitations**

This study was performed in mice and did not determine whether MECP2 directly affects transcription of the genes identified.

**Impact**

Phosphorylation of MECP2 affects its ability to regulate gene expression and fibrosis. Strategies to inhibit MECP2 might be developed for treatment or prevention of liver fibrosis.

In the context of a self-limiting injury and acute inflammation, fibrogenesis is an important contributor to wound repair and regeneration. The purpose of fibrogenesis is to form a temporary extracellular matrix (ECM)–rich barrier known as granulation tissue, which serves to maintain tissue integrity and prevent infection. Once inflammation subsides and effective regenerative processes are underway, fibrogenesis subsides and gives way to fibrolysis, leading to the natural breakdown of temporary granulation tissue and its replacement with repaired epithelial and endothelial structures. Where an injury and/or inflammation persists or if regeneration is impaired, such as in the aging organ, then fibrogenesis fails to subside and instead promotes the net deposition and maturation of fibril-forming collagen-rich ECM. In time, if unabated, nonresolving fibrogenesis leads to the formation of highly crosslinked mature scar tissue that distorts and perturbs normal organ architecture and function. Liver fibrosis is a common pathologic process associated with the majority of chronic liver diseases and, in the absence of an effective treatment for the underlying cause of liver damage, will often progress to end-stage cirrhosis and/or hepatocellular carcinoma.^1^, ^2^ The relatively recent discovery that fibrogenesis is highly dynamic, with the potential to both regress and progress, was an important conceptual milestone, as was the clinical observation made across multiple types of liver disease that fibrosis can spontaneously regress upon effective therapeutic removal of the causative agent. These discoveries have stimulated new investigations into the molecular mechanisms of fibrogenesis and have convinced the pharmaceutical industry that fibrosis is an attractive and tractable therapeutic target in chronic liver diseases.

A further conceptual milestone was the experimental demonstration that, irrespective of cause of liver injury, myofibroblasts are the central cellular drivers of collagen deposition.^1^, ^3^ Liver myofibroblasts are rare in uninjured liver, but in response to damage and inflammation are generated chiefly from resident cells of mesenchymal lineage, the major sources being hepatic stellate cells (HSCs) and periportal fibroblasts.^4^ Upon hepatocellular damage, peri-sinusoidal HSCs are triggered to undergo a complex phenotypic conversion (or transdifferentiation) into α–smooth muscle actin–positive (α-SMA^+^), collagen-secreting myofibroblasts.^1^, ^2^, ^3^, ^4^ Where injury resolves, HSC-derived myofibroblasts are either removed from the tissue by apoptosis or may undergo a partial reversion of phenotype to return to a non–collagen-expressing state.^5^ Experimental in vivo studies strongly support the concept that myofibroblasts are required for fibrogenesis to take place and that their subsequent removal is necessary for cessation of fibrogenesis and a mechanistic switch to fibrolysis. Furthermore, although HSCs are normally quiescent, upon transdifferentiation, they enter the cell cycle and, in response to paracrine and autocrine mitogens (eg, the platelet-derived growth factor PDGF-BB), adopt a highly proliferative and migratory state that serves to amplify and spread the fibrogenic reaction.^4^ Hence, the complex events that enable HSC transdifferentiation and the mechanisms by which the cell adopts its proliferative and profibrogenic behaviors are of major interest in the goal of therapeutic targeting of liver fibrosis.

We previously proposed that HSC transdifferentiation is under tight epigenetic control and identified the prototypic methyl-DNA binding protein Mecp2 as being essential for experimental liver fibrosis.^6^ Subsequently, a similar role for Mecp2 has been described in the bleomycin model of lung fibrosis and in the development of interstitial fibrosis in a mouse model of myocardial infarction, and other reports have confirmed profibrogenic functions for Mecp2 in myofibroblasts from multiple tissue origins.^7^, ^8^, ^9^ In our earlier studies, we focused on Mecp2 as a key transcriptional repressor of the nuclear hormone receptor PPAR-γ because down-regulation of PPAR-γ is a necessary event for HSCs to adopt a fully transdifferentiated myofibroblast state.^6^ Other proposed targets of Mecp2 relevant to its role in fibrosis include the tuberous sclerosis proteins TSC1 and TSC2, which regulate fibroblast differentiation and proliferation; the hedgehog receptor PTCH1 (Patched 1); HDAC6 (histone deacetylase 6); DUSP5 (dual specificity phosphatase 5); the Wnt pathway regulator SFRP4 (secreted frizzled-related protein 4); transforming growth factor (TGF) β1–induced α-SMA; and RASAL1 (RAS GTPase activating-like protein 1). In this study, we used bulk transcriptome profiling to determine that Mecp2 exerts broad influence over the coding and noncoding transcriptional landscapes of the HSC-derived myofibroblast and is required for the expression of transcripts that control DNA integrity and replication. Given the importance of these observations, we also identified a site-specific phosphorylation event on the Mecp2 protein that is required for its stimulation of HSC proliferation and that, upon mutation, reduces the level of toxin-induced liver fibrosis compared with that of wild-type (WT) mice. This work therefore expands our understanding of the mechanisms by which Mecp2 regulates myofibroblast behavior and shows a regulated translational modification of the protein that may be targeted for antifibrotic purposes.

## Materials and Methods

### Ethics

The authors hold appropriate licenses for animal experiments, which were issued/approved by local ethics committee and UK Home Office.

### Animal Strains and Experimental In Vivo Liver Fibrosis Models

WT strain C57Bl/6 and *Mecp2*^*–/y*^ mice (strain B6.129P2[C]-Mecp2tm1.1Bird/J) were obtained from The Jackson Laboratory (Bar Harbor, ME). *Mecp2*^*S80A*^ mice were a gift from Qiang Chang, (University of Wisconsin, Madison, WI).^10^

### Acetaminophen (Paracetamol) Acute Injury

Eight-week-old male Mecp2^S80A^ and WT littermate mice were injected intraperitoneally with *N*-acetyl-ρ-aminophen (APAP) at 500 mg/kg body weight. APAP (Sigma-Aldrich, St Louis, MO) was dissolved in 0.6 mL of warm (37°C) sterile phosphate-buffered saline before injection. Animals were killed at 4 hours after APAP administration, after which serum and liver tissue were collected.

### Acute Carbon Tetrachloride Model

Eight- to 10-week-old male Mecp2^S80A^ and WT littermate mice were intraperitoneally injected with a single dose of a carbon tetrachloride (CCl_4_)/olive oil mixture in a 1:1 (volume/volume) ratio at 2 μL per gram of body weight. Tissues were harvested at 24, 48, and 72 hours after CCl_4_ injection. At least 4 animals were used per treatment group.

### Chronic Carbon Tetrachloride Model

Mice were intraperitoneally injected twice weekly for 4 weeks with a mixture of CCl_4_/olive oil in a 3:1 (volume/volume) at 2 μL per gram of body weight. Tissues were harvested at 24 hours after the final CCl_4_ injection.

### Cell Isolation and Culture

Mouse hepatic stellate cells from C57Bl6 WT, *Mecp2*^–/y^*,* or *Mecp2*^*S80A*^ livers were isolated using sequential pronase/collagenase digestion followed by density-gradient centrifugation with Nycodenz (Axis Shield, UK) (11% over 16.5%) as described by Mann et al.^6^ Two to 5 livers were pooled for each HSC isolation and considered as n = 1. Purity of mouse HSC preparations was assessed by autofluorescence 1 day after isolation and was found to be >97%. Isolation of hepatocytes and Kupffer cells was performed as previously described by Perugorria et al.^11^ Hepatocytes were cultured in William’s medium E (Sigma-Aldrich) supplemented with 10% fetal bovine serum. Mouse HSCs and Kupffer cells were cultured on plastic in Dulbecco’s modified Eagle medium, supplemented with 100 units/mL penicillin, 100 µg/mL streptomycin, 2 mmol/L l-glutamine, and 16% fetal calf serum. Cell cultures were maintained at 37°C at an atmosphere of 5% CO_2_. Freshly isolated HSCs (day 0) were considered quiescent and were cultured in plastic dishes to transdifferentiate into activated HSC (day 7 onward).

### Small Interfering RNA Transfection and Hyaluronic Acid Quantification

Mouse WT HSCs were prepared from 3 separate isolations. Primary mouse HSCs (1 × 10^6^/well) were seeded in 6-well plates and transfected at day 5 with small interfering RNA (siRNA) by using INTERFERin siRNA Transfection Reagent (Polyplus Transfection, Illkrich, France). The siRNAs used were *Stealth* siRNA targeting Has2 (hyaluronan synthase 2) messenger RNA (mRNA) or *Stealth* Negative Control Duplex siRNA (Invitrogen, Waltham, MA). The final concentration of siRNA was 50 nmol/L. The cells were allowed to grow for 72 hours after transfection; then they were harvested and RNA isolated. Conditioned media was collected for HA quantification using the Hyaluronan Quantikine enzyme-linked immunosorbent assay kit (DHYAL0; R&D Systems, Minneapolis, MN) per the manufacturer’s instructions.

For quantitative reverse-transcription polymerase chain reaction (qRT-PCR) analysis, 3-(4,5-dimethylthiazol-2-yl)-2,5-diphenyltetrazolium bromide (MTT) assay, bromodeoxyuridine (BrdU) assay, coding-noncoding gene coexpression network analysis, histology/immunohistochemistry, sodium dodecyl sulfate/polyacrylamide gel electrophoresis, immunoblotting, preparation and sequencing of WT and S80Ki whole-liver RNA samples, transcript quantification and differential expression analyses, and microarray and computational analyses, see the Supplementary Materials and Methods.

### Statistical Analysis

Data are expressed as mean ± standard error of the mean (SEM). *P* values were calculated by either analysis of variance with Bonferroni post hoc test or by Student *t* test as appropriate.

## Results

### Mecp2 Regulates Transcripts Controlling Myofibroblast DNA Replication and Integrity, Metabolism, and Fibrogenesis

**Figure 1:**
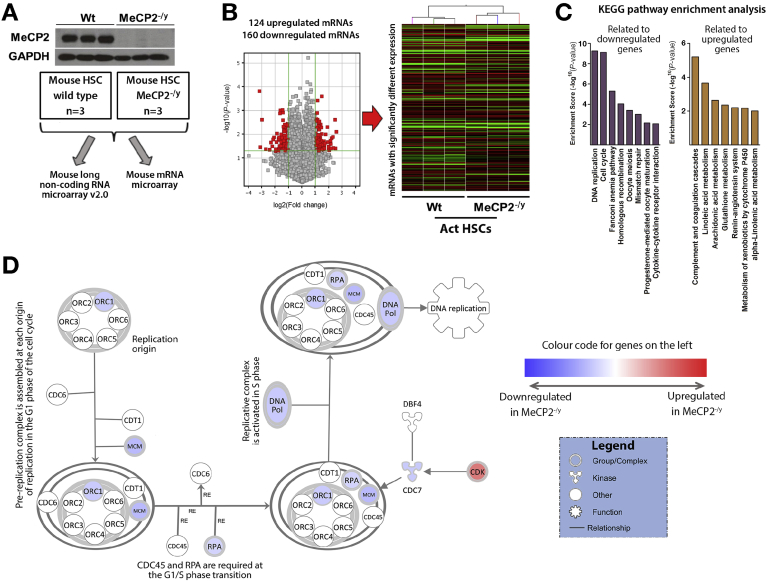
Mecp2 regulates the transcripts controlling myofibroblast DNA replication, cell cycle and integrity, metabolism, and fibrogenesis. (*A*) Western blot for Mecp2 in HSCs from WT or *Mecp2*^*–/y*^ mice. Schematic showing the samples used for lncRNA microarray and RNA microarray. (*B*) Volcano plot and heatmap displaying results of mRNA microarray performed on activated HSCs isolated from 3 control and 3 *Mecp2*^*–/y*^ mice. (*C*) Representation of the groups with 2-fold or greater change in gene expression after the KEGG pathway enrichment analysis. (*D*) Top significantly enriched canonical pathway identified by IPA, showing cell cycle control of chromosomal replication. Blue nodes signify down-regulated gene Mecp2^–/y^ fibroblasts; red signifies up-regulation. Symbol shapes signify the nature of the encoded protein, and unshaded symbols signify genes relevant to the pathway but not differentially expressed in our data set.

**Figure 2:**
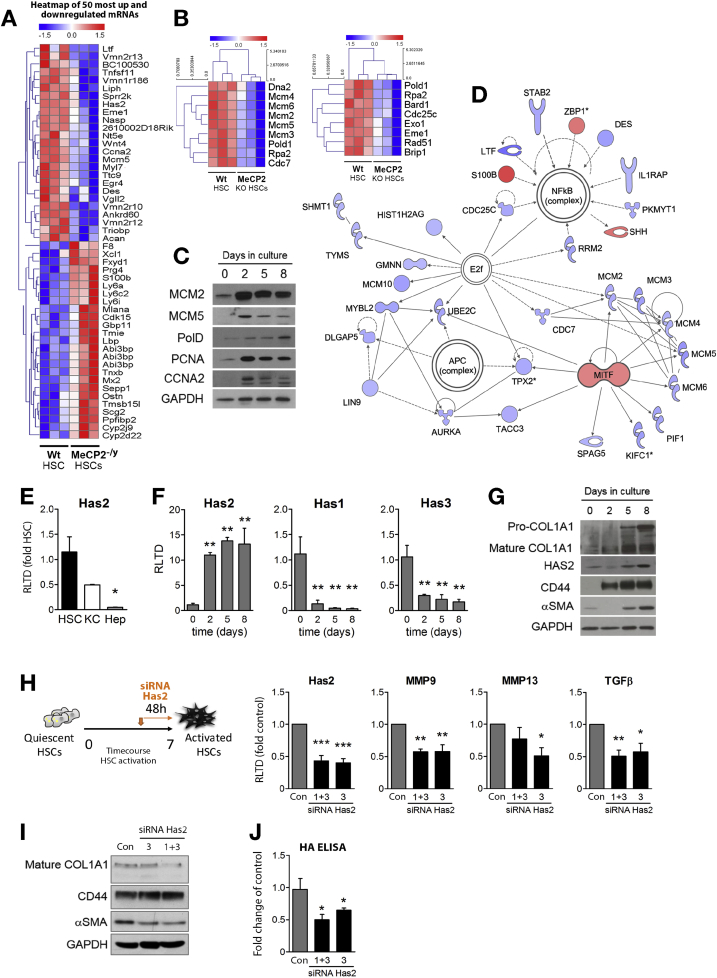
Mecp2 regulates DNA replication and repair systems; Has2 is involved in HSC transdifferentiation. (*A*) Heatmap displaying the results of mRNA microarray performed on activated HSCs isolated from 3 control and 3 *Mecp2*^*–/y*^ mice. The top 50 most up-regulated and down-regulated genes are shown. Blue denotes down-regulation; red, up-regulation; white, unchanged. (*B*) Heatmap displaying the most down-regulated genes with a fundamental role in DNA repair responses (*C*) Western blot for MCM2, MCM5, POLD1, PCNA, CCNA2, and GAPDH in WT HSCs after 0, 2, 5, and 8 days in culture. (*D*) IPA was used to form a network of focus genes that are downstream targets of differentially expressed mRNAs. Blue nodes signify that a gene was down-regulated in Mecp2^*–*/y^ myofibroblasts; red signifies up-regulation. Symbol shapes signify the nature of the encoded protein, and unshaded symbols signify genes relevant to the pathway but not differentially expressed in our data set. (*E*) mRNA level of Has2 from HSCs, Kupffer cells (KC), and hepatocytes (Hep). (*F*) mRNA levels of Has2, Has1, and Has3 in HSCs after 0, 2, 5, and 8 days in culture. (*G*) Western blot for COL1A1, HAS2, the receptor CD44, α-SMA, and GAPDH in HSCs after 0, 2, 5, and 8 days in culture. (*H*) Schematic showing the silencing experiment: HSCs were cultured for 5 days, then transfected with a cocktail of Has2 targeting siRNA (1 + 3), single Has2 targeting siRNA (3) or a negative control (Con) and harvested 48 hours later on day 7. mRNA levels of Has2, MMP9, MMP13, and transforming growth factor β in control and Has2 siRNA–transfected HSCs. Error bars represent mean ± SEM. Statistical significance was determined by ANOVA; *P < .05, **P < .01, ***P < .001. (*I*) Western blot for Col1A1, CD44, α-SMA, and GAPDH and (*J*) HA enzyme-linked immunosorbent assay in HSCs transfected with Has2 siRNA (as in *H*).

**Supplementary Figure 1:**
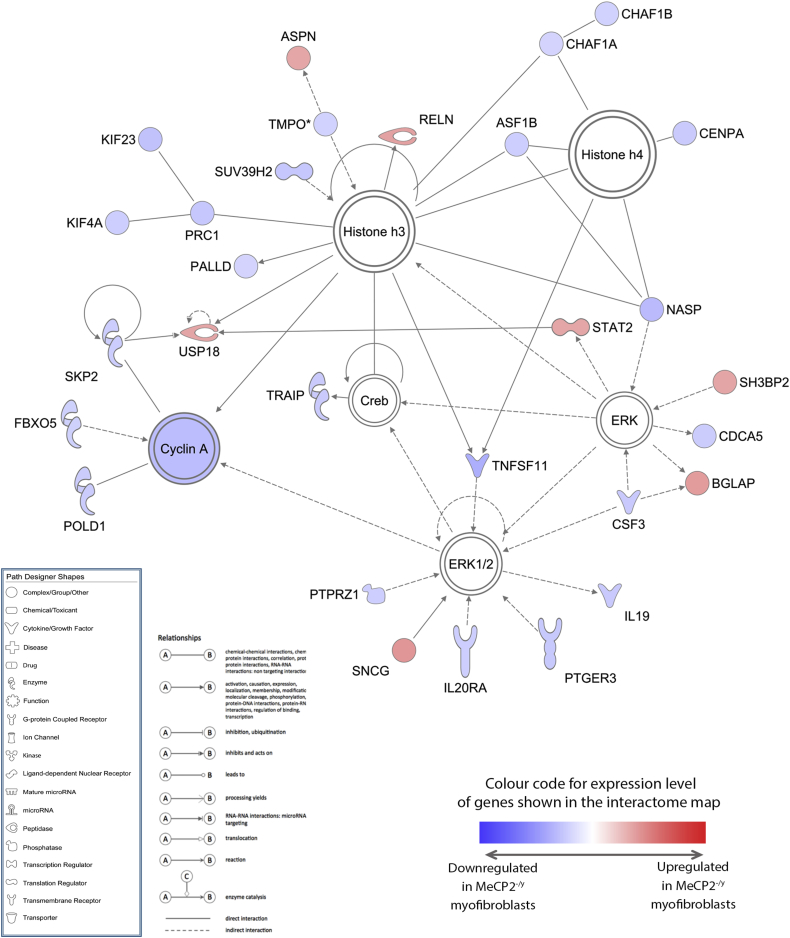
One of the networks of differentially expressed focus genes formed by IPA. Blue nodes signify that a gene was down-regulated in MeCP2^–/y^ fibroblasts; red indicates up-regulation. Symbol shapes signify the nature of the encoded protein, and unshaded symbols signify genes relevant to the pathway but not differentially expressed in our data set.

**Supplementary Figure 2:**
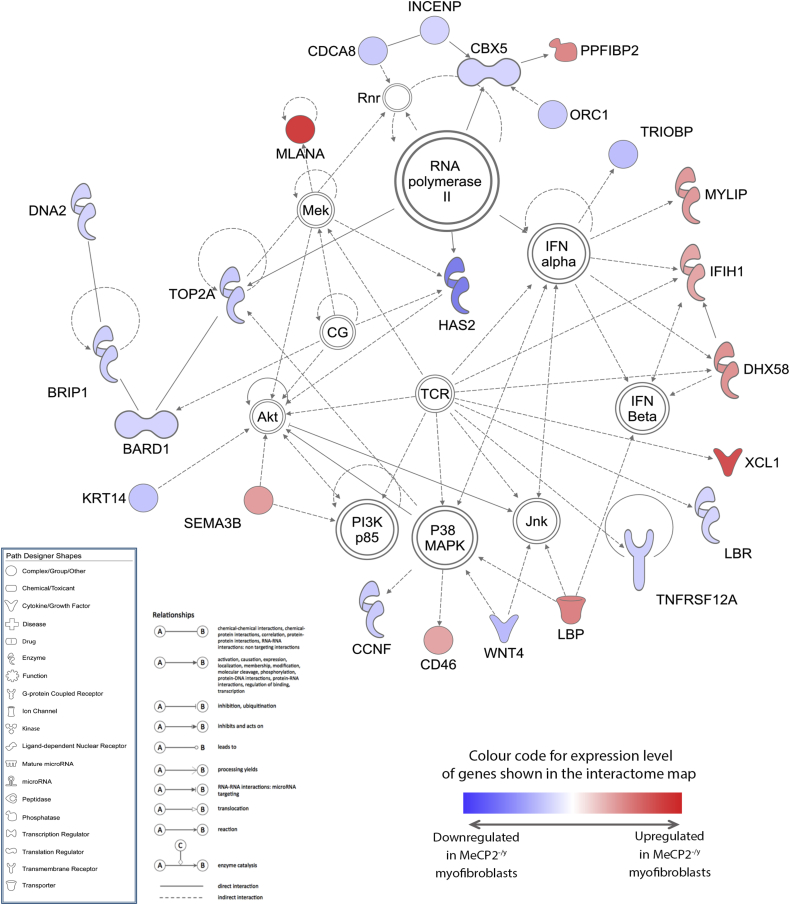
Another network of differentially expressed focus genes formed by IPA. Blue nodes signify that a gene was down-regulated in MeCP2^–/y^ fibroblasts; red indicates up-regulation. Symbol shapes signify the nature of the encoded protein, and unshaded symbols signify genes relevant to the pathway but not differentially expressed in our data set.

**Supplementary Figure 3:**
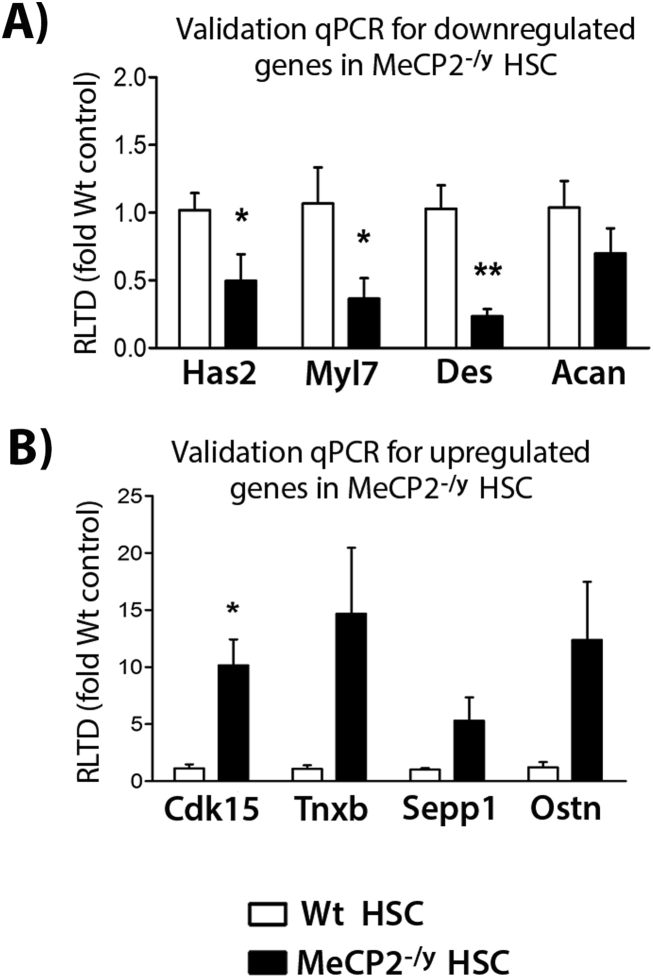
(*A*) mRNA levels of Has2, Myl7, desmin (Des), and aggrecan (Acan) as down-regulated genes and (*B*) Cdk15, Tnxb, Sepp1, and Osnt as up-regulated genes were quantified by qRT-PCR to validate the results of microarray. Error bars in relevant panels represent mean ± SEM. Statistical significance was determined by Student *t* test; *P < .05, **P < .01. RLTD, relative level of transcriptional difference.

To determine the biochemical pathways under the control of Mecp2, we carried out a Mouse LncRNA Array v2.0 (8 × 60,000; Arraystar, Rockville, MD) RNA microarray screen on primary WT and *Mecp2*^*–/y*^ murine HSCs that had undergone culture-induced myofibroblast transdifferentiation. The rationale for selection of array rather than RNA sequencing for our transcriptome analysis was a desire to map Mecp2-regulated long noncoding transcripts (lncRNAs) as well as mRNAs. Typically, lncRNAs are expressed at considerably lower abundance than mRNAs and can be overwhelmed and underreported by RNA sequencing.^12^ Use of the sequence-specific probe hybridization approach of the microarray platform avoids this bias and enables a broader coverage of transcripts to be quantified. Figure 1*A* confirms the absence of expression of the Mecp2 protein in 3 individual *Mecp2*^*–/y*^ HSC-derived myofibroblast lines used for transcriptome analysis. Figure 1*B* shows a volcano plot and associated heatmap (*right panel*) displaying 124 up-regulated and 160 down-regulated mRNA species identified from the microarray as reporting a 2-fold or greater change in expression between *Mecp2*^*–/y*^ and WT myofibroblasts (Supplementary Tables 1 and 2). Kyoto Encyclopedia of Genes and Genomes (KEGG) pathway analysis (Figure 1*C*) showed that with loss of Mecp2, there is reduced expression of mRNAs encoding proteins involved in DNA replication, cell cycle control, DNA damage response (eg, Fanconi anemia pathway), and cytokine-chemokine receptor interactions. In contrast, up-regulated mRNAs in *Mecp2*^*–/y*^ myofibroblasts encode proteins involved in complement and coagulation cascades, metabolism of linoleic acid, arachidonic acid, glutathione, xenobiotics (cytochrome P450s), and control of the renin-angiotensin system. Ingenuity Pathway Analysis (IPA) of the differentially expressed genes further confirmed a role for Mecp2 in the control of DNA replication by showing a number of genes involved in the pathways that were down-regulated in *Mecp2*^*–/y*^ myofibroblasts (Figure 1*D*). Furthermore, IPA analysis also shows a major role for Mecp2 in the overall epigenetic/transcriptional state of chromatin in myofibroblasts, with histones H3 and H4 as well as RNA polymerase II identified as the central targets affected by the changes in gene expression in *Mecp2*^*–/y*^ myofibroblasts (Supplementary Figures 1 and 2). To validate these data, we randomly selected transcripts from the top 25 most overexpressed and underexpressed genes in *Mecp2*^*–/y*^ myofibroblasts for qRT-PCR analysis (Figure 2*A*). Has2, Myl7 (myosin light chain 7), and desmin transcripts were validated as underexpressed relative to the WT; by contrast, a fourth transcript (aggrecan) failed validation (Supplementary Figure 3*A*). All 4 mRNAs (Cdk15 [cyclin dependent kinase 15], Tnxb [tenascin XB], Sepp1 [selenoprotein P], and Ostn [osteocrin]) selected from the heatmap of overexpressed transcripts were validated by qRT-PCR (Supplementary Figure 3*B*). We conclude that the observed de novo expression of Mecp2 protein that occurs early in HSC transdifferentiation affects the expression of approximately 280 protein-coding transcripts that influence key regulatory pathways relevant to fibrosis.

### Mecp2 Regulates DNA Replication and Repair Systems

**Supplementary Figure 4:**
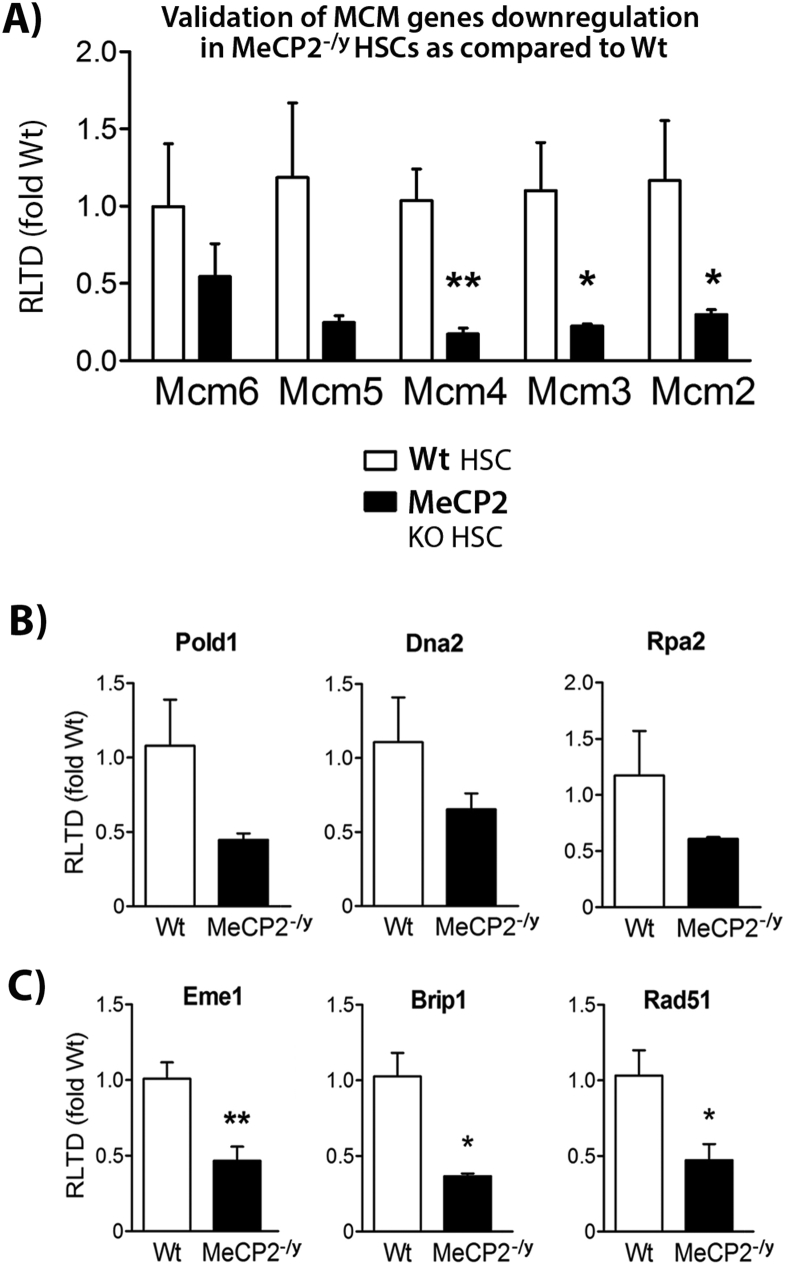
(*A*) mRNA level of Mcm6-2 quantified by qRT-PCR to validate the results of microarray. (*B*, *C*) mRNA levels of Pold1, Dna2, Rpa2, Eme1, Brip1, and Rad51 quantified by qRT-PCR to validate the results of microarray. Error bars in relevant panels represent mean ± SEM. Statistical significance was determined by Student *t* test; *P < .05, **P < .01. KO, knockout; RLTD, relative level of transcriptional difference.

We previously observed that myofibroblasts derived from *Mecp2*^*–/y*^ HSCs are difficult to establish in culture, and there are many reports documenting the pro-proliferative properties of Mecp2 in HSCs and other fibroblast lineages.^13^, ^14^, ^15^, ^16^ It was therefore of particular interest that KEGG pathway analysis highlighted a role for Mecp2 in the control of DNA replication (Figure 1*C*). IPA analysis of these data shows that a number of down-regulated genes in *Mecp2*^*–/y*^ HSCs are in the DNA damage and DNA replication pathways (Figure 1*D*). Closer interrogation of the array data showed reduced expression levels of several subunits for the minichromosome maintenance protein complex (MCM), which is a DNA helicase essential for DNA replication (Figure 2*B*, *left panel*). In addition, Cdc7 (cell division cycle 7-related protein kinase), a kinase regulator of MCM, was also expressed at reduced levels, as was a second DNA helicase, Dna2 (DNA replication adenosine triphosphate–dependent helicase/nuclease) and the single-stranded DNA-binding protein Rpa2 (replication protein A2) (Figure 2*B*). Given the critical role of the Mcm complex in DNA replication, we validated these data by qRT-PCR, which confirmed reduced expression of transcripts for MCM2–6 subunits in *Mecp2*^*–/y*^ HSCs (Supplementary Figure 4*A*). Similarly, qRT-PCR confirmed reduced expression of mRNAs encoding PolD1 (polymerase delta 1), Dna2, and Rpa2 in Mecp2-deficient cells (Supplementary Figure 4*B*). To show the relevance of these findings to HSC transdifferentiation, MCM2, MCM5, and PolD1 proteins were all induced at an early stage of HSC transdifferentiation (culture day 2) and in concert with induction of CCNA2 (cyclin A2), which promotes transition through G1/S and G2/M, and the DNA polymerase processivity factor PCNA2 (proliferating cell nuclear antigen 2), which is a marker for DNA replication (Figure 2*C*). A further striking observation was an apparent role for Mecp2 in the control of genes involved in the Fanconi anemia pathway, which play a fundamental role in DNA repair responses. IPA analysis showed that multiple genes in this pathway display reduced transcript expression in *Mecp2*^*–/y*^ HSCs (Figure 2*D*, down-regulated genes shown in blue), and random selection of 3 of these genes (Eme1 [essential meiotic structure-specific endonuclease 1], Brip1 [BRCA1-interacting protein 1], and Rad51) provided qRT-PCR validation of diminished expression (Supplementary Figure 4*C*).

As an exemplar for the potential for using the Mecp2 transcriptome map to identify novel regulators of liver fibrosis, we selected Mecp2-regulated Has2 for further investigation. We confirmed that Has2 mRNA is highly expressed in HSC-derived myofibroblasts relative to Kupffer cells and hepatocytes (Figure 2*E*). Furthermore, Has2 transcript expression is elevated during culture-induced HSC transdifferentiation, but by contrast, its functionally related family members Has1 and Has3 were transcriptionally down-regulated (Figure 2*F*). Induction of Has2 protein was observed to occur with similar kinetics as Col1A1 (type IA1 collagen) and α-SMA; in addition, immunoblotting for the HA receptor CD44 showed a similar de novo induction occurring within 2 days of culturing (Figure 2*G*). It has previously been reported that the hyaluronan synthase isoform Has2 plays a profibrogenic function in the lung; however, its role as a profibrogenic factor in HSC was not determined.^17^ Therefore, we used siRNA-mediated knockdown of Has2 in transdifferentiating HSC and observed an associated 50% reduction in expression of TGFβ1 and blunted expression of transcripts for the ECM modulators matrix metalloproteinase (MMP) 9 and MMP13 (Figure 2*H*). We further observed a reduction in Col1A1 and α-SMA expression (Figure 2*I*), as well as a reduction in HA production in HSCs transfected with Has2 siRNA (Figure 2*J*). These data indicate that Mecp2 can extend its control over fibrogenesis beyond that of its immediate target genes.

### Mecp2 Is a Regulator of the Noncoding Transcriptome

**Figure 3:**
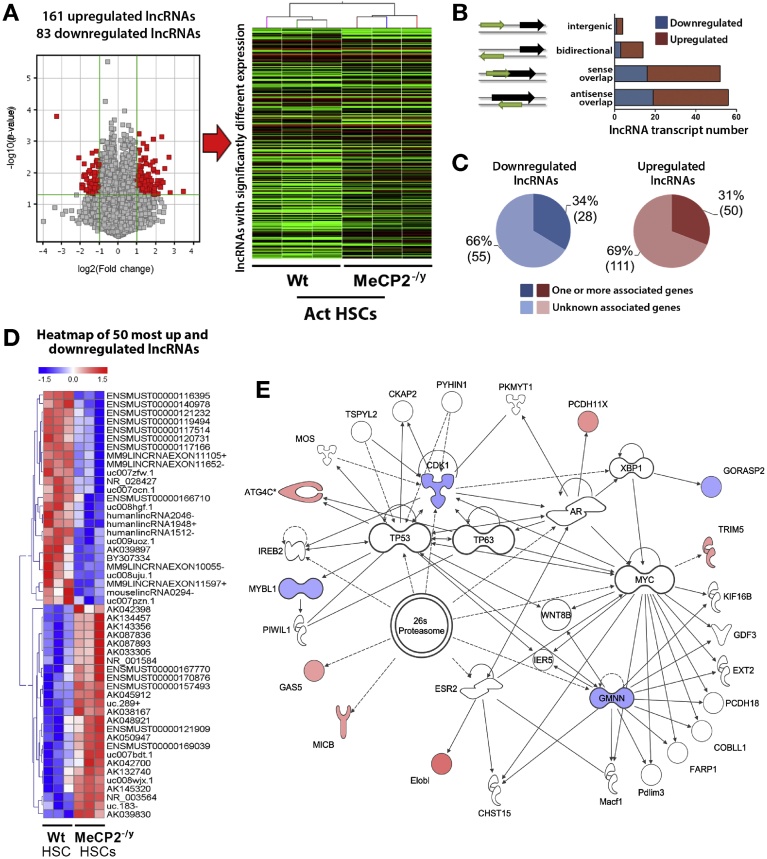
Mecp2 is a regulator of the noncoding transcriptome. (*A*) Volcano plot and heatmap displaying results of lncRNA microarray performed on activated HSCs isolated from 3 control and 3 *Mecp2*^*–/y*^ mice. (*B*) Schematic showing the lncRNAs classified according with their genomic location. (*C*) Schematic showing the lncRNAs according to their association with known and unknown protein coding genes. (*D*) Heatmap displaying results of lncRNA microarray performed on activated HSCs isolated from 3 control and 3 *Mecp2*^*–/y*^ mice. The top 50 most up-regulated and down-regulated lncRNAs are shown. Blue are down-regulated; red, up-regulated; white, unchanged. (*E*) IPA was used to form a network of focus genes that are downstream targets of differentially expressed lncRNAs. Blue nodes show down-regulated genes in Mecp2^–/y^ myofibroblasts, red nodes are up-regulated. Symbol shape signifies the nature of the encoded protein, and unshaded symbols signify genes relevant to the pathway but not differentially expressed in our data set.

**Supplementary Figure 5:**
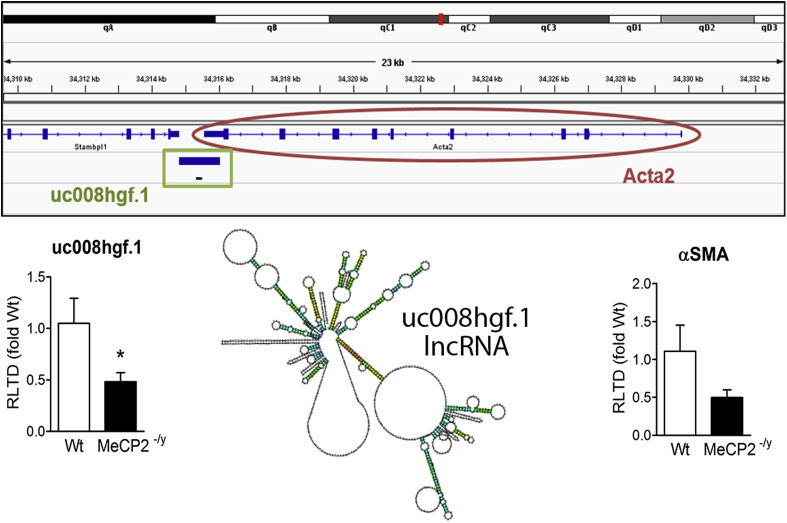
Identification/representation of the down-regulated transcript *uc008hgf* as in close physical association with the ACTA2 (α-SMA) locus. mRNA levels of uc008hgf.1 and α-SMA quantified by qRT-PCR to validate the results of microarray. Predicted RNA secondary structures from uc008hgf.1 lncRNA obtained with CentroidFold software using the CONTRAfold model. Error bars in relevant panels represent mean ± SEM. Statistical significance was determined by Student *t* test; *P < .05.

**Supplementary Figure 6:**
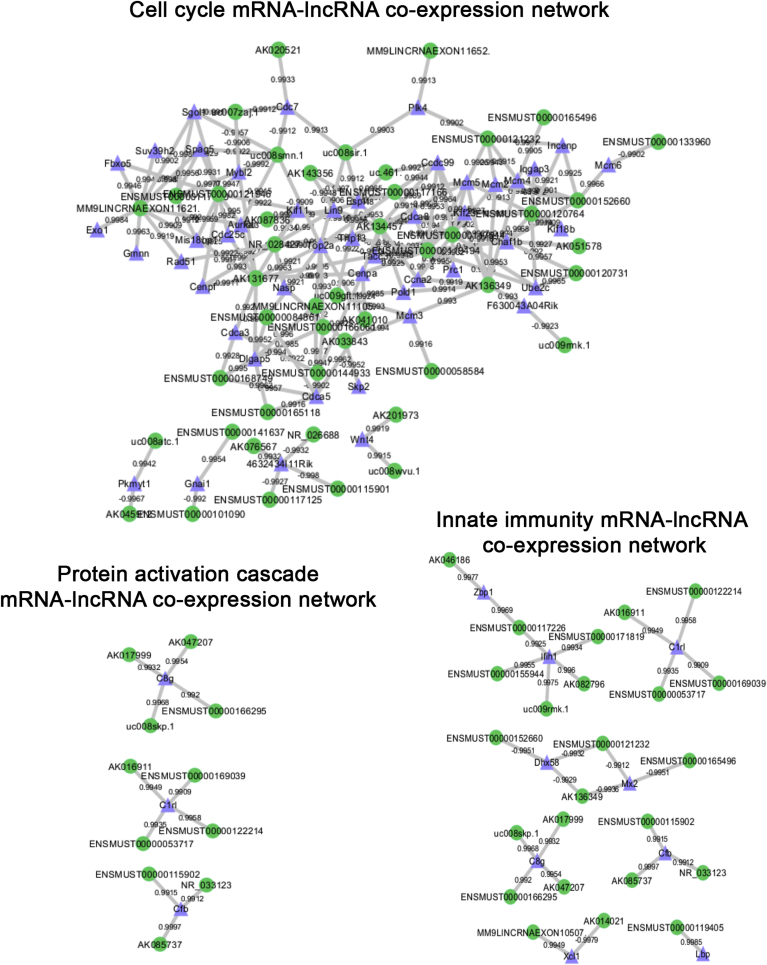
Graphical view of differentially expressed coding-noncoding gene coexpression network analysis represents similar patterns of expression and interconnectivity of the most representative Gene Ontology terms (cell cycle, DNA metabolic process, innate immunity, and protein activation cascades). The interaction network was produced in Cytoscape, where nodes correspond to mRNA (*triangles in purple*) or lncRNA (*circles in green*), and the edges are the coexpression links.

We next focused our attention on the potential for Mecp2 to influence the HSC lncRNA landscape using the array data from our analysis of WT and *Mecp2*^*–/y*^ cells. A volcano plot of lncRNAs detected in the array and subsequent heat mapping of differentially regulated transcripts showed that absence of Mecp2 alters the expression of 244 lncRNAs, of which 161 species were up-regulated and 83 were down-regulated relative to the WT (Figure 3*A* and Supplementary Tables 3 and 4). lncRNAs can be classified according to their genomic location. Mecp2 had the greatest influence on lncRNAs derived from intragenic (so-called *lincRNAs*) sense and antisense overlapping localities and had a lesser effect on lncRNAs classified as intergenic or bidirectional (Figure 3*B*). Further genomic analysis showed that Mecp2 influences the expression of lncRNAs that are classified according to their association with known and unknown protein coding genes (Figure 3*C*). From a focused heatmap of the 50 most up- and down-regulated lncRNAs in *Mecp2*^*–/y*^ cells, (Figure 3*D*), we identified the down-regulated transcript *uc008hgf* as being in close physical association with the Acta2 (α-SMA) locus (Supplementary Figure 5). qRT-PCR confirmed reduced expression of *uc008hgf* and Acta2 in *Mecp2*^*–/y*^ HSCs (Supplementary Figure 5). Coding-to-noncoding coexpression analyses and IPA analysis from the array data identified Mecp2-regulated mRNA-lncRNA networks, including systems controlling DNA metabolic processes, innate immunity, protein activation cascades, and cell cycle (Figure 3*E* and Supplementary Figure 6), thus reaffirming a regulatory role for Mecp2 in DNA replication and repair systems.

### Mecp2 Phosphorylation Is Required for Hepatic Stellate Cell Proliferation and Collagen Expression

**Figure 4:**
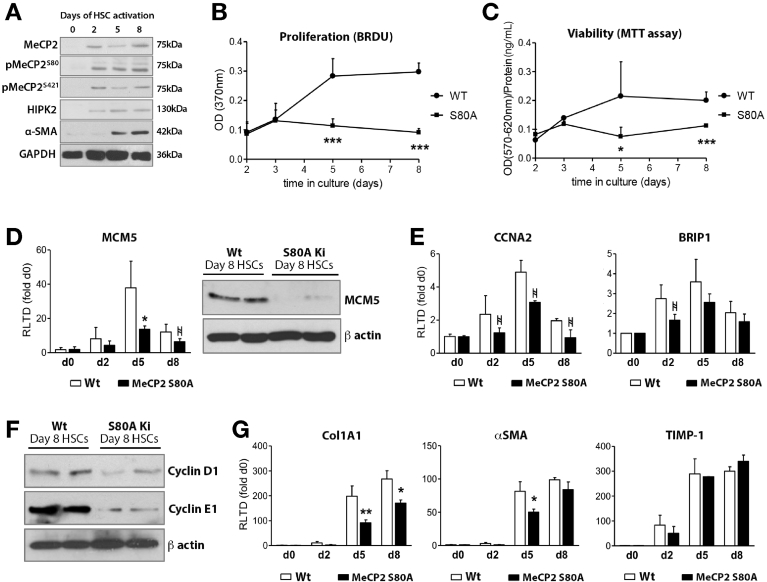
Mecp2 phosphorylation at S80 is required for HSC proliferation and collagen expression. (*A*) Western blot for Mecp2, pMecp2^S80^, pMecp2^S421^, HIPK2, α-SMA, and GAPDH in HSCs after 0, 2, 5, and 8 days in culture. (*B*) Proliferation assay: BrdU incorporation was measured in WT and Mecp2^S80A^ HSCs after 0, 2, 5, and 8 days in culture. (*C*) Viability assay: reduced MTT was measured in Mecp2^S80A^ HSCs (compared with WT HSCs) after 0, 2, 5, and 8 days in culture. (*D*) mRNA level of Mcm5 quantified by qRT-PCR in WT and Mecp2^S80A^ HSCs after 0, 2, 5, and 8 days in culture. Western blot for MCM5 and β-actin in WT and Mecp2^S80A^ HSCs after 8 days in culture. (*E*) mRNA level of CCNA2 and BRIP1 in WT and Mecp2^S80A^ HSCs after 0, 2, 5, and 8 days in culture. (*F*) Western blot cyclin D1 and E1 in WT and Mecp2^S80A^ activated HSCs (8 days in culture). (*G*) Col1A1, α-SMA, and TIMP1 quantified by qRT-PCR in WT and Mecp2^S80A^ HSCs after 0, 2, 5, and 8 days in culture. Error bars represent mean ± SEM. Statistical significance was determined by ANOVA or Students *t* test; *P < .05, ** *P* < .01, *** *P* < .001.  denotes a trend with *P* < .1.

**Supplementary Figure 7:**
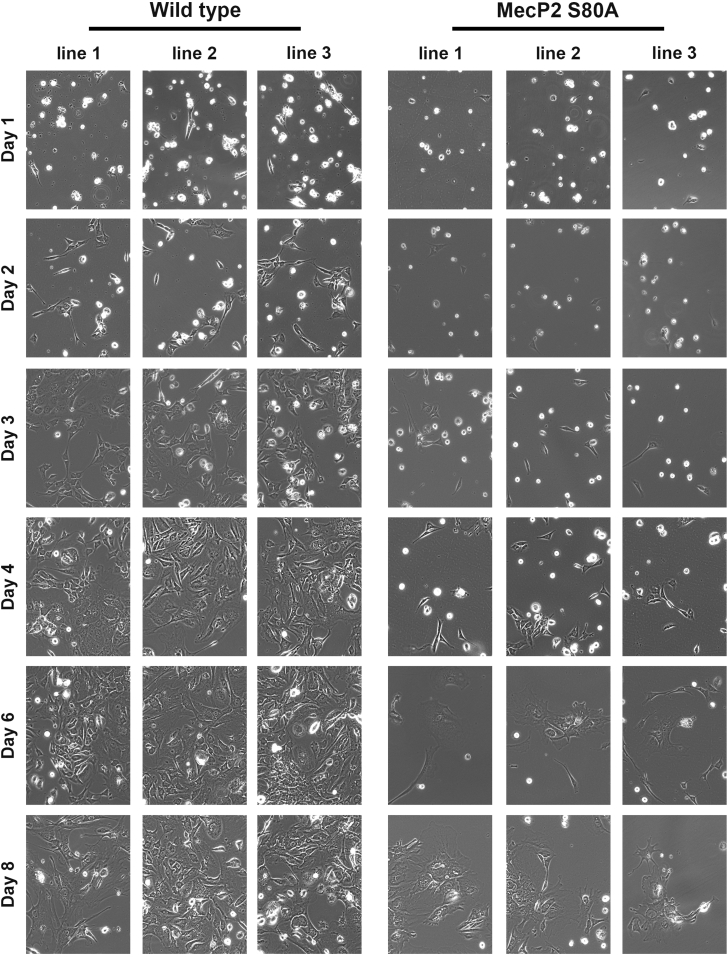
HSCs were isolated from 3 control and 3 *MeCP2*^*S80AKi*^ mice, seeded at equal density at the start of the experiment (day 0), and allowed to transdifferentiate in vitro. Photomicrographs were taken on days 1, 2, 3, 4, 6, and 8. Representative pictures from all 3 lines from WT and *MeCP2*^*S80AKi*^ are shown.

With the longer-term aim of designing molecular strategies for therapeutically manipulating the fibrogenic activities of Mecp2, we examined its posttranslational modification by site-specific phosphorylation. Mass spectrometry analysis has identified at least 8 sites of phosphorylation, including S80, T148/S149, S164, S229, S399, S421, and S424.^18^ Mutations at S80 and S421/S424 have been studied in neurons and shown to modify Mecp2 function.^18^ Of particular importance was the discovery that phosphorylation at the S80 residue is important for DNA binding, such that mutating S80 to alanine reduced the affinity of Mecp2 for multiple target gene promoter sequences.^10^ Immunoblotting for Mecp2 and its phospho-modified forms was carried out across a time course of culture-induced HSC transdifferentiation. As anticipated from our previous work, Mecp2 controls an epigenetic pathway that promotes myofibroblast transdifferentiation and fibrosis.^6^ Mecp2 was absent in freshly isolated (day 0) HSCs but was induced during the early (initiation) phase of transdifferentiation, appearing at day 2 of culture, and this expression persisted throughout further culturing (Figure 4*A*). Of note, the molecular weight of Mecp2 detected in HSCs was approximately 70 kDa, which has previously been reported as the weight of the slower-migrating phosphorylated form of the protein.^19^ Antibodies recognizing phosphorylation at S80 (pMecp2^S80^) and S421 (pMecp2^S421^) also detected a protein of approximately 70 kDa that was absent in freshly isolated HSCs and appeared at day 2 of culture. Furthermore, we also detected at the same time point induction of HIPK2 (homeodomain-interacting protein kinase 2), which is reported to bind to Mecp2 and phosphorylate it at its S80 residue.^20^ These data encouraged us to examine the role of pMecp2^S80^ in HSCs using primary cells isolated from digested livers of mutant Mecp2^S80A^ knock-in (S80A) mice. Although we were able to establish Mecp2^S80A^ HSC lines, they were morphologically observed to be more rounded (ie, less activated) and were slower growing than WT lines (Supplementary Figure 7); the reduced proliferative rate was confirmed by BrdU incorporation (Figure 4*B*), and MTT assays were carried out across a time course of transdifferentiation (Figure 4*C*). Moreover, S80A HSCs were impaired for expression of Mcm5 transcript and protein (Figure 4*D*) and displayed diminished expression of CCNA2 and Brip1 (Figure 4*E*). In addition, day 8 S80A HSCs expressed considerably less cyclin D1 and E1 (Figure 4*F*). Given our previous observation that Mecp2 is required for HSC expression of fibrogenic genes, we next determined if phosphorylation at S80 was required for this function. As shown in Figure 4*G*, S80A HSCs consistently expressed reduced levels of transcripts for COL1A1 and displayed a delay in maximal expression of α-SMA; however, induction of TIMP1 (tissue inhibitor of metalloproteinase-1) mRNA was no different between S80A and WT HSC. These data suggest that S80 phosphorylation selectively modulates a subset of the fibrogenic characteristics of HSC.

### Mecp2-S80 Phosphorylation Is Required for Toxin-Induced Liver Fibrosis

**Figure 5:**
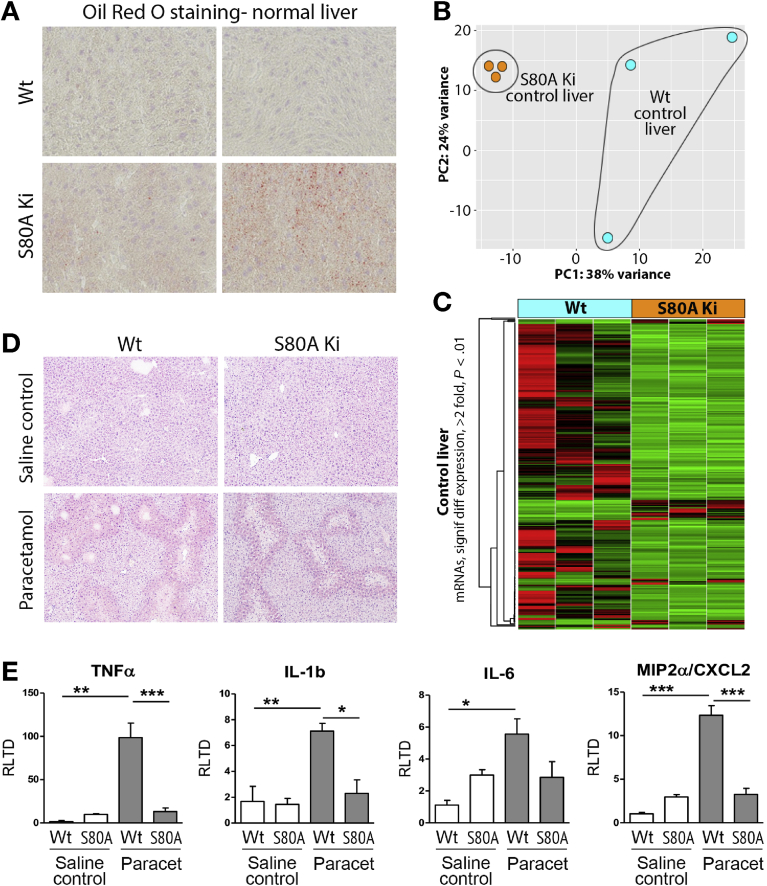
Mecp2^S80^ phosphorylation causes baseline difference in gene expression in uninjured liver and is required for paracetamol-induced liver fibrosis. (*A*) Liver sections showing neutral triglycerides and lipids staining with Oil Red O from WT and Mecp2^S80A^ mice. (*B*) Gene expression variances between WT and Mecp2^S80A^ uninjured livers displayed as PCA plot of scaled log2-transformed transcript counts. (*C*) Heatmap displaying results of transcriptome sequencing carried out using 3 uninjured livers from WT and Mecp2^S80A^ mice. The genes with greater than 2-fold change and significant to *P* < .01 are shown. Green are down-regulated in Mecp2^S80A^; red, up-regulated; black, unchanged. (*D*) Liver morphology shown with H&E staining from WT and Mecp2^S80A^ mice, control or treated with paracetamol. Photomicrographs are at original magnification of ×100. (*E*) mRNA levels of tumor necrosis factor α, IL-1β, IL-6, and MIP2α/CXCL2 quantified by qRT-PCR in WT and Mecp2^S80A^ mice, control or treated with paracetamol (n = 8 per group). Error bars in relevant panels represent mean ± SEM. Statistical significance was determined by ANOVA or Student *t* test; *P < .05, ** *P* < .01, *** *P* < .001. signif diff, significantly different.

**Figure 6:**
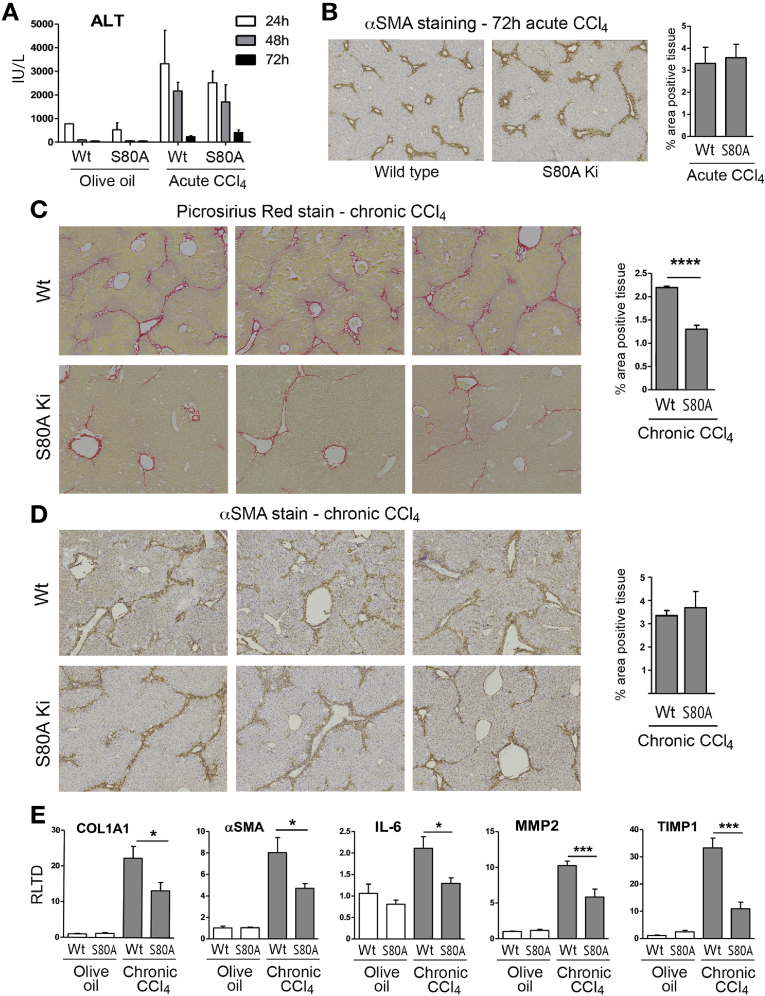
Mecp2^S80^ phosphorylation is required for CCl_4_-induced liver fibrosis. (*A*) Alanine transaminase (ALT) assessment at 24, 48, and 72 hours after CCl_4_/olive oil injection in WT and Mecp2^S80A^ mice treated with acute CCl_4_ or vehicle. (*B*) Histologic sections showing α-SMA staining from WT and Mecp2^S80A^ mice treated with acute CCl_4_. Graph shows the percentage of the area positive for α-SMA staining (n = 6 per group). (*C*, *D*) Liver sections showing collagen staining (Picrosirius Red) and α-SMA from WT and Mecp2^S80A^ mice treated with chronic CCl_4_ or olive oil vehicle. Associated graphs show the percentage of the area positive for Picrosirius Red or α-SMA staining. Photomicrographs are at original magnifications of ×40. (*D*) mRNA level of COL1A1, α-SMA, IL-6, MMP2, and TIMP1 quantified by qRT-PCR in WT and S80A mice treated with olive oil vehicle or chronic CCl_4_. Error bars in relevant panels represent mean ± SEM. Statistical significance was determined by ANOVA or Student t test; ∗*P*<.05, ∗∗*P*<.01, ∗∗∗*P*<.001. Signif diff, Significantly different.

**Figure 7:**
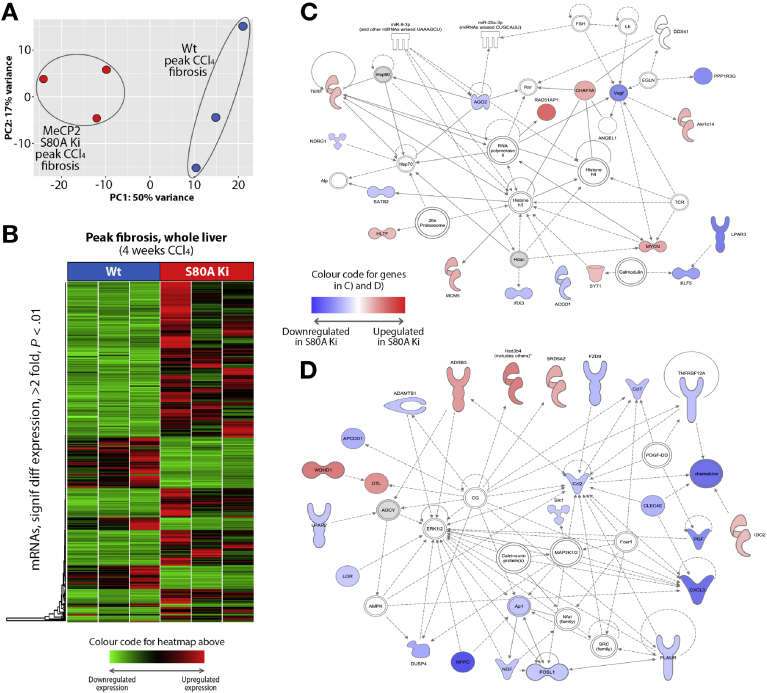
Mecp2^S80^ phosphorylation regulates gene expression in CCl_4_-induced liver fibrosis. (*A*) Gene expression variances between WT and Mecp2^S80A^ fibrotic livers displayed as PCA plots of scaled log2-transformed transcript counts. (*B*) Heatmap displaying results of transcriptome sequencing carried out using 3 CCl_4_-injured livers from each of the WT and Mecp2^S80A^ mice; genes with greater than 2-fold change and significant to *P* < .01 are shown. Green indicates negative values (down-regulated in Mecp2^S80A^); red, positive (up-regulated in Mecp2^S80A^); black, unchanged. (*C*, *D*) IPA was used to form a network of focused genes that are downstream targets of differentially expressed RNAs. Blue nodes show down-regulated genes in Mecp2^S80A^ CCl_4_-injured liver; red nodes are up-regulated. Symbol shapes signify the nature of the encoded protein (see Supplementary Figures 1 and 2 for shape information), and unshaded symbols signify genes relevant to the pathway but not differentially expressed in our data set. signif diff, significantly different.

**Supplementary Figure 8:**
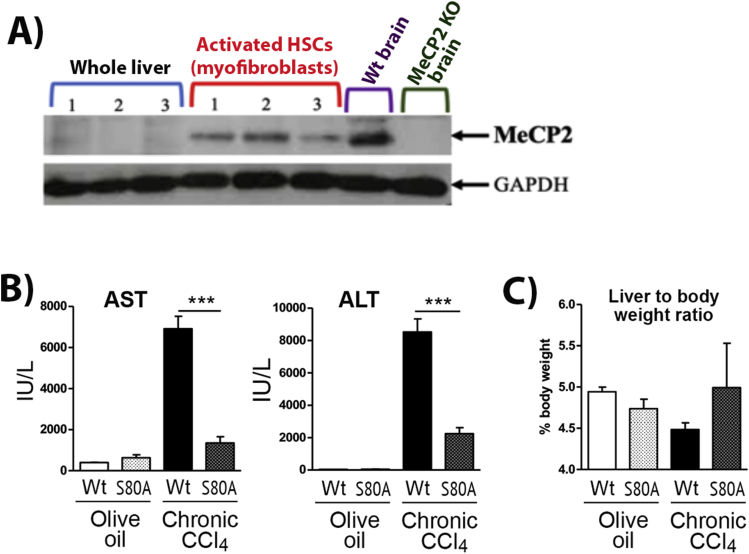
(*A*) Western blot for MeCP2 in whole liver or activated HSCs; MeCP2 WT and KO brain was used as positive or negative control, respectively. (*B*) Liver–to–body weight ratio in WT and MeCP2^S80A^ mice treated with chronic CCl_4_ or vehicle. Error bars in relevant panels represent mean ± SEM. ***P < .001. (*C*) Assessment of serum transaminases alanine transaminase (ALT) and aspartate transaminase (AST). KO, knockout.

We were next interested to determine whether modulation of HSC behavior by S80 phosphorylation affects liver fibrosis. Before determining a role in fibrosis, we asked if the S80A mutation has any effect on the normal physiology and response of the liver to acute toxic injury. Histologic examination of S80A livers did not show any gross anatomic abnormalities; however, we did note a mild steatosis, which was confirmed by Oil Red-O staining (Figure 5*A*). This observation was followed up with transcriptomic sequencing of uninjured WT and S80A Knockin livers, which showed segregated clustering of 2 groups, as determined by using principal component analysis (PCA) (Figure 5*B*), suggesting that S80A livers have phenotypic differences at baseline. A heatmap generated from the transcriptome analysis shows 31 up-regulated and 233 down-regulated RNA species reporting a 2-fold or greater change in expression between S80A compared with WT control livers (Figure 5*C* and Supplementary Table 5). We observed no gross differences in the response of S80A mice to sublethal paracetamol intoxication (Figure 5*D*), although examination of inflammatory gene expression did show unanticipated defects in the induction of tumor necrosis factor α, interleukin (IL) 1β, IL-6, and CXCL2 (chemokine ligand 2) in paracetamol-injured S80A livers (Figure 5*E*). Given these effects of the S80A mutation on liver physiology and response to acute injury, we reassessed the cellular expression of MeCP2 in mouse liver. MeCP2 protein was barely detectable by Western blotting in whole liver but was abundantly expressed in HSCs (Supplementary Figure 8*A*); this result supports previous reports from our group and other investigators that hepatocytes express very low levels of MeCP2, whereas activated HSCs abundantly express the protein.^21^, ^22^, ^23^ Similar to injury with paracetamol, acute damage with CCl_4_ was similar between S80A and the WT based on measurement of alanine aminotransferase (Figure 6*A*). Moreover, analysis of α-SMA expression as a surrogate for HSC transdifferentiation showed a similar acute fibrogenic response between S80A and the WT (Figure 6*B*). By contrast, chronic injury with CCl_4_ suggested partial protection of S80A mice from development of fibrosis as determined by Picrosirius red staining and morphologic quantification of cross-linked collagens (Figure 6*C*), although no difference was observed in the amount of α-SMA staining (Figure 6*D*). This protective effect was mirrored by reduced hepatic expression of COL1A1, α-SMA, IL-6, MMP2, and TIMP1 (Figure 6*E*). In addition, we measured improved liver function (aspartate aminotransferase and alanine aminotransferase) and liver/body weight measurements as outputs of liver health in the S80A mutant mice (Supplementary Figure 8*B* and *C*). Of note, similar chronic CCl_4_ challenge in Mecp2^S421A/S424A^ mice showed no effect on fibrosis or liver function (data not shown). We conclude that site-specific phosphorylation of Mecp2 at its S80 residue is required for a robust fibrogenic reaction to iterative liver damage but is not required for normal liver physiology or for the wound-healing response to acute liver damage. Given the protective effect of S80A point mutation on fibrosis, we performed transcriptomic sequencing of CCl_4_-injured livers. Sequencing data from 2 groups were normalized and used to generate a PCA plot, which showed clear phenotypic differences between the WT and S80A (Figure 7*A*). Interrogation of the sequencing data showed reduced expression of 116 genes and up-regulation of 306 genes in S80A livers compared with the WT (Figure 7*B* and Supplementary Table 6). IPA analysis of differentially expressed genes in S80A-injured livers showed similar networks to those identified in *Mecp2*^*–/y*^ myofibroblasts, particularly pathways involving histones H3 and H4, extracellular signal–regulated kinases 1 and 2, and RNA polymerase II (Figure 7*C* and *D* and Supplementary Figures 1 and 2). These data suggest a role for S80 phosphorylation in controlling Mecp2-mediated regulation of chromatin structure and gene transcription as well as mitogen-activated protein kinase signaling cascades in hepatic myofibroblasts and liver fibrosis.

## Discussion

Experimental in vivo studies in mice across multiple organ systems (liver, lung, and heart) have identified Mecp2 as an important epigenetic profibrogenic factor.^6^, ^14^ These data are supported by in vitro studies suggesting that Mecp2 promotes the fibrogenic characteristics of fibroblasts and myofibroblasts from numerous tissues.^7^, ^8^, ^9^ An exception to these observations was a recent report of Mecp2 suppressing the fibrogenic characteristics of human dermal fibroblasts, which remains to be validated and show an impact on fibrosis.^7^ Hence, a consensus is emerging that in the majority of organs, Mecp2 promotes fibrosis through its activities in the fibroblast/myofibroblast. Of relevance to the functions of MeCP2 in liver fibrosis, expression of MeCP2 is found at very low levels in healthy liver,^21^, ^22^ and this was confirmed in our study. By contrast, MeCP2 expression is elevated in fibrotic liver where its expression is predominantly localized to liver myofibroblasts.^23^ Here, we shed further light on the degree to which Mecp2 exerts its influence on the phenotype of HSC-derived myofibroblasts by discovering that it modulates the expression of at least 529 transcripts, comprising 284 mRNAs and 244 lncRNAs. Differential transcript expression in *Mecp2*^*–/y*^ HSCs included up- and down-regulation in both mRNA and lncRNA families. Although Mecp2 was originally described as a gene silencer,^24^, ^25^ more recent studies have described cell-type–specific functions for Mecp2 and an unexpected role as a gene activator.^26^, ^27^, ^28^ These dual opposing functions of Mecp2 are at least in part explained by the activities of its numerous coregulators; for example, CREB (cAMP response element binding) enhances transcription, whereas HDAC/Sin3A (histone deacetylase/paired amphipathic helix protein) complexes that assemble with Mecp2 at the promoters of repressed genes are known to have potent suppressive activities on transcription.^29^ Additionally, Mecp2 can influence gene expression via its role in chromatin organization and architecture, which is further indicated in the data presented in this report.^30^, ^31^, ^32^ We have also described how Mecp2 can indirectly influence transcription by modulating the expression of other epigenetic factors such as ASH1 (histone-lysine *N*-methyltransferase) and EZH2 (enhancer of zeste homolog 2) that associate with regulatory regions of profibrogenic genes.^6^, ^33^ Furthermore, as we have discovered in this study, Mecp2 has a regulatory influence on lncRNAs, which, in large part, exert their regulatory effects via chromatin remodeling activities. These alternative mechanisms to the DNA-binding functions of Mecp2 highlight an important limitation of the work presented here to be addressed in future: our available data cannot distinguish the degree to which Mecp2 controls the HSC transcriptional landscape through its direct actions at gene promoters vs its various indirect modes of action. A further limitation is the use of cultured HSCs, which may not accurately recapitulate changes in gene expression associated with HSC transdifferentiation in vivo.^34^ However, a danger with isolating transdifferentiated HSCs from the damaged liver is the degree to which subsets of phenotypically distinct cells are preferentially recovered by the isolation protocol. Furthermore, this may be exacerbated in *Mecp2*^*–/y*^ livers where fibrosis is minimal; because *Mecp2*^*–/y*^ HSCs have an altered phenotype, they are more challenging to identify and isolate from the damaged liver. Despite these limitations, by comparing the transcriptional landscapes of WT and *Mecp2*^*–/y*^ HSCs, we have discovered, to our knowledge, genes that were previously unrecognized as Mecp2 regulated and that to date have not been studied in the context of liver fibrosis yet functionally can be linked to the fibrotic process.

HSCs are typically quiescent, but upon transdifferentiation they will proliferate under the stimulation of a variety of mitogenic factors such as PDGF, IL-6, glucose, and leptin.^4^ Initiation of DNA replication is a key regulatory process in HSC transdifferentiation and, as in all proliferating cells, requires “melting” of double-stranded DNA at the origin recognition complex. A key role for the origin recognition complex is to load the MCM2–7 replicative helicase onto the DNA, which, upon completion, results in assembly of the pre-replicative complex that licenses progression to the S phase.^35^ MCM proteins are required at abundant levels in proliferative cells to ensure accuracy of DNA replication and to protect against replicative stress during the S phase.^36^ Indeed, in the absence of a full complement of MCM proteins, genome integrity is lost.^37^ We show here that MCM proteins are induced with HSC transdifferentiation from either low or, in the case of MCM5, undetectable levels of expression in quiescent HSCs. To our knowledge, we have for the first time described Mecp2 as being required for expression of multiple Mcm gene transcripts. These findings suggest a vital role for Mecp2 as a regulator of DNA replication and integrity and explain numerous reports of associations of Mecp2 with cell proliferation.^38^, ^39^ We also report that Mecp2 regulates Has2, which is the major isoform of the HASs expressed in mesenchymal cells where it is responsible for production HA, a key component of the ECM and a modulator of collagen deposition.^40^ HAS2 has also been implicated as a regulator of cell senescence and proliferation, with suppression of HAS2 in tumor cells arresting the cell cycle in the G1 phase.^41^ More recently, Has2 has been described as promoting the invasive phenotype of fibrogenic lung fibroblasts and, when experimentally down-regulated in these cells, promotes senescence and resolution of pulmonary fibrosis.^17^ We found that HAS2 and HA receptor CD44 are induced during HSC transdifferentiation. Of note, CD44 protein expression was undetectable in quiescent HSCs and was abundantly expressed at day 2 of culture, which coincided with abundant induction of MCM2 and MCM5 proteins and cyclin A2 (*CCNA2*), the latter being required for the G2/M phase transition. We therefore propose that Mecp2 regulates initiation and progression of HSC DNA replication via 2 distinct pathways. Molecular targeting of Mecp2 may therefore offer an attractive approach for suppressing the proliferative and invasive behavior of fibrogenic cells. To this end, we were encouraged by the finding that HSCs lacking the ability to phosphorylate the S80 residue of Mecp2 were defective for expression of MCM5 and displayed impaired proliferation. Because S80A mutant mice were able to mount a normal acute wound repair response to toxic liver injury yet were at least partially protected from fibrosis in the context of chronic liver damage, targeting translational modification events on Mecp2 may offer a therapeutic strategy in fibrosis. As previously mentioned, in the absence of fibrosis, MeCP2 is expressed in the liver at low to negligible levels. Moreover, MeCP2 is reported to be expressed in hepatocytes, and specific deletion of epithelial MeCP2 promotes steatosis.^42^ We also observed mild steatosis in the livers of S80A knockin animals and elevated expression of inflammatory mediators in S80A knockin liver in response to acute injury with paracetamol. Hence, although S80 phosphorylation in myofibroblasts offers an intriguing therapeutic target, the same modification may be important for baseline metabolic functions of MeCP2 in hepatocytes, and this would need to be considered if S80 or its kinase HIPK2 were to be developed for therapeutic applications.

In summary, Mecp2 helps orchestrate widespread changes in the RNA landscape that convert the quiescent HSC into fibrogenic cells and is required for the expression of genes that regulate entry and progression of HSC DNA replication. Because site-specific phosphorylation of Mecp2 contributes to these events and is a modulator of liver fibrosis, manipulation of enzymes regulating Mecp2 phosphorylation has potential for therapeutic targeting.

## Supplementary Materials and Methods

### Quantitative Reverse-Transcription Polymerase Chain Reaction Analysis

Total RNA was purified from isolated cells by using the Total RNA purification kit (Qiagen, Manchester, UK) following the manufacturer’s instructions. One microgram of total RNA was DNAse treated (Promega, Madison, WI) and used as a template to generate complementary DNA by using a random hexamer primer (p[dN]6) and MMLV reverse transcriptase (Promega). Primer sequences are included in Supplementary Table 1. SYBR Green qRT-PCR reactions were performed in a total volume of 13 μL containing 20 ng of complementary DNA template, 6.5 μL of SYBR Green JumpStart Taq ReadyMix (Sigma-Aldrich), and 20 pmol of forward and reverse primers (Supplementary Table 1, Supplementary Table 2, Supplementary Table 3). The PCR reaction was carried out on a 7500 Fast Real-Time PCR System (Applied Biosystems, Warrington, UK) with the following parameters: 1 cycle at 95 C for 10 seconds followed by 40 cycles at 95°C for 10 seconds, 57°C′–60°C (for primer pair–specific annealing temperatures, see Supplementary Table 1) for 30 seconds, and finally 72°C for 30 seconds. Melt curve analysis was used to confirm presence of a single PCR product. All reactions were normalized to β-actin or GAPDH internal control, and relative level of transcriptional difference calculated by using the following equation: (1/[2*A*]) ×100.

### MTT Assay

Cellular viability was determined by using the MTT assay where day 2, 3, 5, and 8 primary HSCs from WT or *MeCP2*^*S80A*^ livers were seeded onto 24-well plates and treated with MTT (5 mg/mL, Sigma) for 1.5 hours. Cells were rinsed with phosphate-buffered saline and fixed in isopropanol; the plates were shaken at 240 revolutions for minute for 10 minutes. The absorbance was measured at 570/620 nm in a SpectraMax (Grodig, Austria) plate reader.

### BrdU Assay

Cells were labeled with BrdU in fresh cell media containing 16% fetal bovine serum for 18 hours. Cell proliferation was determined by BrdU incorporation, following the manufacturer’s instructions for the Cell Proliferation enzyme-linked immunosorbent assay (Roche, Basel, Switzerland).

### Coding-Noncoding Gene Coexpression Network Analysis

To identify possible biological functions, we performed a coding-noncoding gene coexpression network analysis^1^ of the coexpressed lncRNAs related to genes. The coexpression matrix was calculated with the Perl package “Statistics Basic,” and the “correlation” function was filtered to include only the interactions for which the absolute value of the Pearson correlation coefficient *r* was > 0.99.^2^, ^3^ The coding-noncoding coexpression network was visualized with the bioinformatics software Cytoscape.^4^

### Histology and Immunohistochemistry

Formalin-fixed, paraffin-embedded tissue sections were stained with 0.1% Picrosirius red and H&E with established protocols. Immunohistochemistry was performed on deparaffinized sections, and endogenous peroxidase activity was blocked by using 0.6% hydrogen peroxide/methanol solution. Heat-mediated antigen retrieval was achieved by using antigen unmasking solution. Endogenous avidin and biotin were blocked with an Avidin/Biotin Blocking Kit (Vector Laboratories, Burlingame, CA), and nonspecific binding was blocked by using 20% swine serum for 30 minutes; then α-SMA 1:1000 (F3777; Sigma-Aldrich) was incubated overnight at 4°C. The next day, slides were washed and incubated with biotinylated goat anti-fluorescein 1:300 (BA-0601, Vector Laboratories, Peterborough, UK) for 1 hour. Slides were washed and incubated with Vectastain Elite ABC Reagent for 45 minutes. Staining was visualized by using a 3,3′-diaminobenzidine tetra hydrochloride peroxidase substrate kit and counterstained with Mayer’s hematoxylin before mounting. Slides were analyzed at 20× magnification with a Nikon (Tokyo, Japan) Eclipse Upright microscope and NIS-Elements BR analysis software (Nikon). Ten random fields of liver were analyzed per section. All images presented are at original magnifications of ×100, and scale bars equal 100 μm unless otherwise stated.

### Oil Red O Staining

Fifteen-micrometer frozen sections were fixed in Baker’s formol-calcium, washed in distilled water, and then incubated with 60% isopropanol for 5 minutes. Sections were then incubated in Oil Red O solution for 20 minutes, transferred to 60% isopropanol for 5 minutes, washed with distilled water, and then mounted with Kaiser’s glycerol-gelatin.

### Sodium Dodecyl Sulfate–Polyacrylamide Gel Electrophoresis and Immunoblotting

Whole-cell extracts were prepared, and protein concentration of samples was determined by using Bio-Rad DC Protein Assay (Bio-Rad, Watford, UK). Proteins were separated on 10% or 12% sodium dodecyl sulfate polyacrylamide gel and transferred onto a nitrocellulose membrane in buffer containing 25 mmol/L Tris, 192 mmol/L glycine, and 20% methanol. Blots were blocked with Tris-buffered saline/Tween 20 (0.1%) containing 5% milk protein before overnight incubation with primary antibodies: rabbit anti-MeCP2 (C15410052; Diagenode, Liege, Belgium), MCM2 (D7G11; Cell Signaling Technology, Danvers, MA), MCM5 (ab75975; Abcam, Cambridge, UK), PolD (ab186407, Abcam), CCNA2 (ab38, Abcam), PCNA (ab2426, Abcam), Col1A1 (AB765P; Millipore, Billerica, MA), α-SMA (A5228, Sigma-Aldrich), HAS2 (sc365263; Santa Cruz Biotechnology, Dallas, TX), CD44 (AF6127, R&D Systems), pMeCP2 S80 (AP3595a; Abgent, San Diego, CA), pMeCP2 S421 (PA5-35396; Thermo Fisher Scientific, Waltham, MA), HIPK2 (5091, Cell Signaling Technology), and rabbit anti-GAPDH (ab22555, Abcam). Secondary antibody anti-rabbit (7074S, Cell Signaling Technology) and anti-mouse (A4416, Sigma-Aldrich) horseradish peroxidase–conjugate antibody was use at 1:2000 dilution. The antibody complexes were detected by chemiluminescence by using Pierce ECL Western Blotting Substrate (Thermo Fisher Scientific).

### Preparation and Sequencing of Wild-Type and S80Ki Whole-Liver RNA Samples

RNA was isolated from WT and S80A Knockin control and peak fibrosis (4 weeks CCl_4_) whole livers. RNA sequencing libraries were created by using Illumina (San Diego, CA) TruSeq stranded mRNA kit. The sequencing was single-end with 75-basepair read length, performed on NextSeq 500 (Illumina) by using a high-output, 75-cycle flow cell, providing >40 million reads per sample.

### Transcript Quantification and Differential Expression Analysis

Sequencing quality was assessed by FastQC software (Available online at http://www.bioinformatics.babraham.ac.uk/projects/fastqc/). Poor-quality bases and adaptors were trimmed using Trimmomatic software,^5^ allowing a maximum of 1 mismatch and a quality threshold of 20. The *quasi-mapping* mode of Salmon^6^ was used for the mapping and quantification of the transcripts. The DESeq2 R package^7^ was used to adjust the read counts by scaling a normalized factor in all libraries and to assess the differential expression of the 2 treatments. The *P* values were corrected by the Benjamini and Hochberg method^8^; then, the expression level was considered significantly different at *P* adjusted of <.05 and log2 fold change of >1. Plots were created with gplots R package. The genes with significantly different expression levels as selected by R were input into the IPA tools (http://www.qiagenbioinformatics.com/products/ingenuity-pathwayanalysis/) for functional annotation.

### Microarray and Computational Analysis

RNA was purified from total RNA after removal of ribosomal RNA (mRNA-ONLY Eukaryotic mRNA Isolation Kit; Epicentre Bio, Madison, WI). Each sample was amplified and transcribed into fluorescent complementary RNA along the entire length of the transcripts without 3′ bias by using a random priming method. The labeled complementary RNAs were purified, and 1 μg of each labeled complementary RNA was hybridized onto the Mouse LncRNA Array v2.0 (8 × 60,000, Arraystar) for 17 hours at 65°C in an Agilent (Santa Clara, CA) Hybridization Oven. A total of 25,376 protein-coding RNAs and 31,423 lncRNAs in the mouse genome were collected from the most authoritative data sources, including RefSeq, University of California–Santa Cruz Knowngenes, Ensembl, and related literature. After the slides were washed, the arrays were scanned by the Agilent DNA Microarray Scanner (part no. G2505C). The acquired array images were analyzed by Agilent Feature Extraction software, version 11.0.1.1. Quantile normalization and subsequent data processing were performed with the GeneSpring GX, version 11.5.1, software package (Agilent). Differentially expressed lncRNAs and mRNAs with statistical significance between the 2 groups were identified through volcano plot filtering. Gene Ontology and Pathway analyses were performed in the standard enrichment computation method. The Integrative Genomics Viewer (https://igv.org/app) was used to visualize and interactively explore the location and organization within the mouse genome of the differentially expressed lncRNAs and their associated protein-coding genes.^9^, ^10^ Predicted secondary structures from lncRNA sequences were obtained using the CentroidFold software, (available online at http://www.ncrna.org/software/centroidfold/) and applying the CONTRAfold model to calculate base-paring probabilities with 2^2^ gamma parameter to control the specificity and sensitivity of predicted base pairs.^11^, ^12^ Genes with expression levels that were considered significantly different were input into the IPA tools (http://www.qiagenbioinformatics.com/products/ingenuity-pathwayanalysis/) for functional annotation.

## References

[1] Hernandez-Gea V., Friedman S.L. (2011). Pathogenesis of liver fibrosis. Annu Rev Pathol.

[2] Lee Y.A., Wallace M.C., Friedman S.L. (2015). Pathobiology of liver fibrosis: a translational success story. Gut.

[3] Puche J.E., Saiman Y., Friedman S.L. (2013). Hepatic stellate cells and liver fibrosis. Compr Physiol.

[4] Tsuchida T., Friedman S.L. (2017). Mechanisms of hepatic stellate cell activation. Nat Rev Gastroenterol Hepatol.

[5] Troeger J.S., Mederacke I., Gwak G.Y. (2012). Deactivation of hepatic stellate cells during liver fibrosis resolution in mice. Gastroenterology.

[6] Mann J., Chu D.C., Maxwell A. (2010). MeCP2 controls an epigenetic pathway that promotes myofibroblast transdifferentiation and fibrosis. Gastroenterology.

[7] He Y., Tsou P.S., Khanna D. (2018). Methyl-CpG-binding protein 2 mediates antifibrotic effects in scleroderma fibroblasts. Ann Rheum Dis.

[8] Nectoux J., Florian C., Delepine C. (2012). Altered microtubule dynamics in Mecp2-deficient astrocytes. J Neurosci Res.

[9] Zhou P., Lu Y., Sun X.H. (2011). Zebularine suppresses TGF-beta-induced lens epithelial cell-myofibroblast transdifferentiation by inhibiting MeCP2. Mol Vis.

[10] Tao J., Hu K., Chang Q. (2009). Phosphorylation of MeCP2 at serine 80 regulates its chromatin association and neurological function. Proc Natl Acad Sci U S A.

[11] Perugorria M.J., Murphy L.B., Fullard N. (2013). Tumor progression locus 2/Cot is required for activation of extracellular regulated kinase in liver injury and toll-like receptor-induced TIMP-1 gene transcription in hepatic stellate cells in mice. Hepatology.

[12] Shi Y., Shang J. (2016). Long noncoding RNA expression profiling using Arraystar LncRNA microarrays. Methods Mol Biol.

[13] Babbio F., Castiglioni I., Cassina C. (2012). Knock-down of methyl CpG-binding protein 2 (MeCP2) causes alterations in cell proliferation and nuclear lamins expression in mammalian cells. BMC Cell Biol.

[14] Tao H., Yang J.J., Shi K.H. (2016). Epigenetic factors MeCP2 and HDAC6 control α-tubulin acetylation in cardiac fibroblast proliferation and fibrosis. Inflamm Res.

[15] Tao H., Yang J.J., Hu W. (2016). MeCP2 regulation of cardiac fibroblast proliferation and fibrosis by down-regulation of DUSP5. Int J Biol Macromol.

[16] Yang J.J., Liu L.P., Tao H. (2016). MeCP2 silencing of LncRNA H19 controls hepatic stellate cell proliferation by targeting IGF1R. Toxicology.

[17] Li Y., Jiang D., Liang J. (2011). Severe lung fibrosis requires an invasive fibroblast phenotype regulated by hyaluronan and CD44. J Exp Med.

[18] Li H., Chang Q. (2014). Regulation and function of stimulus-induced phosphorylation of MeCP2. Front Biol (Beijing).

[19] Bueno C., Tabares-Seisdedos R., Moraleda J.M. (2016). Rett syndrome mutant neural cells lacks MeCP2 immunoreactive bands. PLoS One.

[20] Bracaglia G., Conca B., Bergo A. (2009). Methyl-CpG-binding protein 2 is phosphorylated by homeodomain-interacting protein kinase 2 and contributes to apoptosis. EMBO Rep.

[21] Shahbazian M.D., Antalffy B., Armstrong D.L. (2002). Insight into Rett syndrome: MeCP2 levels display tissue- and cell-specific differences and correlate with neuronal maturation. Hum Mol Genet.

[22] Luikenhuis S., Giacometti E., Beard C.F. (2004). Expression of MeCP2 in postmitotic neurons rescues Rett syndrome in mice. Proc Natl Acad Sci U S A.

[23] Mann J., Oakley F., Akiboye F. (2007). Regulation of myofibroblast transdifferentiation by DNA methylation and MeCP2: implications for wound healing and fibrogenesis. Cell Death Differ.

[24] Klose R., Bird A. (2003). Molecular biology. MeCP2 repression goes nonglobal. Science.

[25] Wakefield R.I.D., Smith B.O., Nan X.S. (1999). The solution structure of the domain from MeCP2 that binds to methylated DNA. J Mol Biol.

[26] Ben-Shachar S., Chahrour M., Thaller C. (2009). Mouse models of MeCP2 disorders share gene expression changes in the cerebellum and hypothalamus. Hum Mol Genet.

[27] Chahrour M., Jung S.Y., Shaw C. (2008). MeCP2, a key contributor to neurological disease, activates and represses transcription. Science.

[28] Sugino K., Hempel C.M., Okaty B.W. (2014). Cell-type-specific repression by methyl-CpG-binding protein 2 is biased toward long genes. J Neurosci.

[29] Della Ragione F., Filosa S., Scalabri F. (2012). MeCP2 as a genome-wide modulator: the renewal of an old story. Front Genet.

[30] Georgel P.T., Horowitz-Scherer R.A., Adkins N. (2003). Chromatin compaction by human MeCP2. Assembly of novel secondary chromatin structures in the absence of DNA methylation. J Biol Chem.

[31] Horike S., Cai S.T., Miyano M. (2005). Loss of silent-chromatin looping and impaired imprinting of *DLX5* in Rett syndrome. Nat Genet.

[32] Nikitina T., Ghosh R.P., Horowitz-Scherer R.A. (2007). MeCP2-chromatin interactions include the formation of chromatosome-like structures and are altered in mutations causing Rett syndrome. J Biol Chem.

[33] Perugorria M.J., Wilson C.L., Zeybel M. (2012). Histone methyltransferase ASH1 orchestrates fibrogenic gene transcription during myofibroblast transdifferentiation. Hepatology.

[34] De Minicis S., Seki E., Uchinami H. (2007). Gene expression profiles during hepatic stellate cell activation in culture and in vivo. Gastroenterology.

[35] Evrin C., Clarke P., Zech J. (2009). A double-hexameric MCM2-7 complex is loaded onto origin DNA during licensing of eukaryotic DNA replication. Proc Natl Acad Sci U S A.

[36] Forsburg S.L. (2004). Eukaryotic MCM proteins: beyond replication initiation. Microbiol Mol Biol Rev.

[37] Bailis J.M., Forsburg S.L. (2004). MCM proteins: DNA damage, mutagenesis and repair. Curr Opin Genet Dev.

[38] Zhao L.Y., Zhang J., Guo B. (2013). MECP2 promotes cell proliferation by activating ERK1/2 and inhibiting p38 activity in human hepatocellular carcinoma HEPG2 cells. Cell Mol Biol (Noisy-le-grand).

[39] Sharma K., Singh J., Frost E.E. (2018). MeCP2 overexpression inhibits proliferation, migration and invasion of C6 glioma by modulating ERK signaling and gene expression. Neurosci Lett.

[40] Albeiroti S., Soroosh A., de la Motte C.A. (2015). Hyaluronan’s role in fibrosis: a pathogenic factor or a passive player?. Biomed Res Int.

[41] Udabage L., Brownlee G.R., Nilsson S.K. (2005). The over-expression of *HAS2, Hyal-2* and CD44 is implicated in the invasiveness of breast cancer. Exp Cell Res.

[42] Kyle S.M., Saha P.K., Brown H.M. (2016). MeCP2 co-ordinates liver lipid metabolism with the NCoR1/HDAC3 corepressor complex. Hum Mol Genet.

