# Supplementary material for: Fibrogenic Activity of MECP2 Is Regulated by Phosphorylation in Hepatic Stellate Cells
Source: Gastroenterology. 2019 Nov;157(5):1398–1412.e9. doi: 10.1053/j.gastro.2019.07.029 (PMC6853276; doi:10.1053/j.gastro.2019.07.029)
Supplement: Supplementary Table 1 [file mmc1.pdf]

Supplementary Table 1. Differentially Expressed upregulated genes that passed the filtering (Fold Change ≥ 2.0, p-value ≤ 0.05)

Accession Number	Gene Symbol	FC	p-val	length	Ch	S	Start	End
NM_001110146	Prg4	5.82	0.008	1884	chr1	-	152296541	152313295
NM_198112	Ostn	5.59	0.039	1268	chr16	+	27307726	27351295
NM_052992	Fxyd1	5.48	0.002	639	chr7	-	31836696	31840675
NM_029993	Mlana	5.00	0.037	461	chr19	+	29772430	29782796
NM_010738	Ly6a	4.95	0.013	842	chr15	-	74825306	74828318
NM_031176	Tnxb	4.56	0.014	9569	chr17	+	34807479	34856760
NM_008510	Xcl1	4.43	0.039	502	chr1	-	166861778	166865641
NM_001099217	Ly6c2	4.41	0.025	875	chr15	-	74938590	74942379
NM_001014422	Abi3bp	4.33	0.018	4454	chr16	+	56477958	56690248
NM_001039647	Gbp11	4.02	0.045	2723	chr5	-	105752044	105775491
NM_020498	Ly6i	3.97	0.032	878	chr15	-	74810241	74813860
NM_178790	Abi3bp	3.91	0.033	4394	chr16	+	56477958	56690248
NM_013606	Mx2	3.90	0.038	2424	chr16	+	97757689	97782506
NM_028979	Cyp2j9	3.74	0.021	1944	chr4	-	96235119	96258176
NM_009115	S100b	3.72	0.005	1676	chr10	+	75716580	75724064
NM_009155	Sepp1	3.33	0.010	2030	chr15	+	3220976	3230508
NM_001033373	Cdk15	3.20	0.024	1477	chr1	+	59313750	59409213
NM_001014399	Abi3bp	3.17	0.039	3884	chr16	+	56477958	56690248
NM_001161374	F8	3.13	0.028	7306	chrX	-	72418055	72625380
NM_019823	Cyp2d22	3.10	0.019	2782	chr15	-	82200956	82210690
NM_146260	Tmie	3.03	0.041	2437	chr9	-	110768549	110782587
NM_009129	Scg2	2.97	0.023	2485	chr1	-	79431243	79436665
NM_008489	Lbp	2.97	0.039	2528	chr2	+	158132228	158158588
NM_207267	Tmsb15l	2.92	0.038	808	chrX	-	133489528	133511390
NM_001163557	Ppfibp2	2.83	0.028	3850	chr7	+	114750357	114892097
NM_172951	Sntg2	2.79	0.010	2337	chr12	-	30859421	31058240
NM_008288	Hsd11b1	2.79	0.000	1381	chr1	-	195047834	195090239
NM_001001446	Cyp2c44	2.76	0.043	1964	chr19	-	44079511	44103737
NM_001102409	Kng2	2.76	0.042	1612	chr16	-	22985924	23029174
NM_001122635	Cdhr4	2.73	0.028	1135	chr9	+	107897371	107902011
NM_001126491	Gm14459	2.72	0.043	540	chrX	-	8091177	8102080
NM_013755	Gyg	2.70	0.048	1739	chr3	-	20021969	20054995
NM_025727	Klhl10	2.69	0.005	2012	chr11	+	100303237	100318338
NM_181728	Art3	2.67	0.047	1578	chr5	+	92760866	92843653
NM_001160386	Dnahc7b	2.66	0.018	12318	chr1	+	46123582	46430395
NM_172524	Nipal4	2.59	0.004	3295	chr11	-	45961656	45979861
NM_008631	Mt4	2.57	0.042	392	chr8	+	96661103	96662931
NM_030150	Dhx58	2.54	0.026	2427	chr11	-	100556197	100565585
NM_001142706	Cfb	2.51	0.014	2763	chr17	-	34993318	34999459
NM_001200041	Gm1078	2.50	0.022	3152	chr7	-	4916861	4922563
NM_007797	Ctla2b	2.50	0.032	856	chr13	-	60996711	60998808
NM_207268	Ccdc87	2.45	0.010	3163	chr19	+	4839365	4842528
NM_001011872	Olfr1034	2.45	0.020	1023	chr2	+	85886600	85887623
NM_009170	Shh	2.44	0.047	2727	chr5	-	28783379	28793641
NM_011430	Sncg	2.44	0.036	757	chr14	-	35183459	35187855
NM_010060	Dnahc11	2.43	0.030	14072	chr12	-	119116454	119437516
NM_024264	Cyp27a1	2.43	0.043	1890	chr1	+	74760147	74784464
NM_001163569	Kif9	2.42	0.028	3162	chr9	+	110379497	110426944
NM_146630	Olfr123	2.41	0.023	930	chr17	+	37932390	37933320
NM_001172055	Bdh2	2.41	0.013	1172	chr3	+	134944184	134967389

NM_018738	Igtp	2.40	0.033	2064	chr11	+	58013057	58021094
NM_139269	Pla2g16	2.40	0.031	3523	chr19	+	7631948	7663035
NM_146015	Efemp1	2.40	0.006	2036	chr11	+	28753204	28826743
NM_027040	1700007K13Rik	2.39	0.003	833	chr2	-	28317520	28321844
NM_053135	Pcdhb10	2.39	0.047	2842	chr18	+	37571327	37574168
NM_177216	Cyb5r2	2.38	0.047	2688	chr7	-	114891968	114901510
NM_013555	Hoxd9	2.38	0.021	2153	chr2	+	74535819	74538265
NM_028169	Fam71e1	2.37	0.041	849	chr7	+	51751957	51756504
NM_008967	Ptgir	2.35	0.010	3334	chr7	+	17491838	17496254
NM_001139519	Zbp1	2.34	0.004	781	chr2	-	173039236	173044423
NM_028623	Cst6	2.34	0.004	3951	chr19	-	5344704	5349574
NM_010358	Gstm1	2.33	0.010	1310	chr3	-	107815167	107820891
NM_153789	Mylip	2.33	0.011	2983	chr13	+	45485110	45507309
NM_001032298	Bglap2	2.31	0.017	471	chr3	-	88181657	88182621
NM_021394	Zbp1	2.30	0.004	1988	chr2	-	173032113	173044423
NM_025759	Speer4d	2.30	0.029	1159	chr5	+	15124916	15129682
NM_008198	Cfb	2.29	0.012	2767	chr17	-	34993318	34999459
NM_178404	Zc3h6	2.29	0.014	4916	chr2	+	128793137	128844299
NM_026880	Pink1	2.26	0.029	2367	chr4	-	137869324	137882211
NM_008486	Anpep	2.26	0.041	3439	chr7	-	86966688	86987238
NM_001042779	Sema3b	2.25	0.015	3305	chr9	-	107500445	107511572
NM_001012322	Sctr	2.23	0.020	1939	chr1	+	121903556	121960109
NM_145467	Itgbl1	2.22	0.000	2357	chr14	+	124059361	124373301
NM_001039122	Defb25	2.21	0.023	297	chr2	-	152448091	152448789
NM_008107	Gdf1	2.21	0.041	1419	chr8	+	72853663	72855487
NM_207657	5031410I06Rik	2.21	0.031	2947	chr5	-	26425207	26431854
NM_013792	Naglu	2.20	0.014	2504	chr11	+	100931407	100938985
NM_010359	Gstm3	2.20	0.018	1202	chr3	-	107766613	107772076
NM_175692	Snhg11	2.20	0.023	5864	chr2	+	158201373	158211881
NM_027153	Pir	2.20	0.002	1601	chrX	+	160707362	160810945
NM_001007570	Slc25a42	2.19	0.044	3181	chr8	-	72708238	72736180
NM_001145926	C530028O21Rik	2.19	0.037	2091	chr6	+	124948627	124953117
NM_011261	Reln	2.18	0.045	11702	chr5	-	21390271	21850523
NM_025496	Cdrt4	2.18	0.048	723	chr11	+	62764694	62806597
NM_026599	Cgnl1	2.16	0.005	6754	chr9	-	71474315	71619409
NM_022880	Slc29a1	2.16	0.001	2047	chr17	-	45722148	45729342
NM_001101475	F830016B08Rik	2.15	0.035	3297	chr18	+	60453033	60462670
NM_012044	Pla2g2e	2.12	0.005	851	chr4	+	138433856	138438729
NM_011322	Scn1b	2.11	0.036	1568	chr7	-	31901542	31911964
NM_175486	6430571L13Rik	2.11	0.004	2552	chr9	+	107242970	107252014
NM_028181	Ccpg1	2.11	0.037	2689	chr9	+	72833357	72863806
NM_001134299	Gm10220	2.11	0.023	2984	chr5	-	26441303	26447965
NM_174857	Mamdc2	2.10	0.041	3338	chr19	-	23377098	23522812
NM_010140	Epha3	2.10	0.011	3978	chr16	-	63545043	63863983
NM_175030	Tctex1d4	2.08	0.033	982	chr4	+	116799417	116801335
NM_001114328	Ccpg1	2.08	0.047	2966	chr9	+	72833357	72861745
NM_001081131	Dhtkd1	2.08	0.002	3535	chr2	-	5819105	5863838
NM_011909	Usp18	2.07	0.046	1778	chr6	+	121195923	121220935
NM_009250	Serpini1	2.07	0.046	3133	chr3	+	75361454	75446445
NM_001136088	Sh3bp2	2.07	0.018	2957	chr5	+	34892336	34906288
NM_029376	Speer4a	2.07	0.036	1503	chr5	-	26360809	26366045
NM_001004159	Clec4b2	2.06	0.035	1044	chr6	+	123123040	123154689
NM_025658	Ms4a4d	2.06	0.026	1480	chr19	+	11611338	11632956
NM_001037713	Xaf1	2.06	0.029	2621	chr11	+	72115130	72127235

NM_010778	Cd46	2.06	0.003	1213	chr1	-	196868093	196918442
NM_181344	C1rl	2.06	0.010	3010	chr6	+	124443130	124460661
NM_028561	Speer4b	2.06	0.027	1977	chr5	-	27822348	27827932
NM_207624	Ace	2.06	0.014	4885	chr11	+	105829260	105851259
NM_030738	Vmn1r65	2.05	0.006	2685	chr7	-	5959351	5962612
NM_025711	Aspn	2.05	0.008	2362	chr13	+	49639811	49662931
NM_027062	C8g	2.04	0.035	1063	chr2	-	25354175	25356026
NM_001081351	A430107O13Rik	2.04	0.039	4898	chr6	+	21935909	22205606
NM_027835	Ifih1	2.04	0.011	5519	chr2	-	62433849	62484312
NM_007796	Ctla2a	2.04	0.030	1373	chr13	-	61035515	61037986
NM_145153	Oas1f	2.03	0.024	1480	chr5	+	121297375	121307995
NM_021486	Bcmo1	2.03	0.034	2327	chr8	+	119619764	119657620
NM_019963	Stat2	2.03	0.024	4402	chr10	+	127707631	127729905
NM_144938	C1s	2.03	0.006	2947	chr6	-	124480361	124492339
NM_001113198	Mitf	2.03	0.014	4890	chr6	+	97757051	97971352
NM_148942	Serpinb6c	2.02	0.020	1390	chr13	-	33971684	33997577
NM_020561	Smpdl3a	2.01	0.006	1791	chr10	+	57514349	57531636
NM_010630	Kifc2	2.01	0.045	3143	chr15	+	76491070	76498626
NM_001040112	Arap1	2.01	0.037	5314	chr7	+	108526635	108561100
NM_008626	Mrc2	2.00	0.037	5801	chr11	+	105153959	105212459
