# Supplementary material for: Fibrogenic Activity of MECP2 Is Regulated by Phosphorylation in Hepatic Stellate Cells
Source: Gastroenterology. 2019 Nov;157(5):1398–1412.e9. doi: 10.1053/j.gastro.2019.07.029 (PMC6853276; doi:10.1053/j.gastro.2019.07.029)
Supplement: Supplementary Table 2 [file mmc2.pdf]

Supplementary Table 2. Differentially Expressed downregulated genes that passed the filtering (Fold Change ≥ 2.0, p-value ≤ 0.05)

Accession number	Gene Symbol	FC	p-val	length	Chr	S	Start	End
NM_009491	Vmn2r10	9.16	0.0002	2544	chr5	-	109424557	109435455
NM_011477	Sprr2k	8.65	0.0340	639	chr3	+	92236503	92237849
NM_001104623	Vmn2r12	7.93	0.0024	2496	chr5	-	109514867	109526883
NM_008216	Has2	6.48	0.0414	4262	chr15	-	56497181	56526101
NM_008522	Ltf	5.40	0.0441	2742	chr9	+	110921795	110945270
NM_001082546	BC100530	5.06	0.0219	453	chr16	-	36359467	36367656
NM_153786	Vgll2	4.68	0.0308	1483	chr10	+	51742491	51748277
NM_022879	Myl7	4.49	0.0161	599	chr11	-	5796639	5798785
NM_007424	Acan	4.33	0.0134	7355	chr7	+	86198368	86259985
NM_011613	Tnfsf11	3.95	0.0498	2243	chr14	-	78677252	78707850
NM_020596	Egr4	3.91	0.0414	2100	chr6	-	85461115	85463536
NM_001167567	Vmn1r186	3.73	0.0203	903	chr7	+	5626923	5627826
NM_001104624	Vmn2r13	3.70	0.0089	2496	chr5	-	109585086	109621126
NM_177752	Eme1	3.54	0.0475	2228	chr11	-	94506315	94515068
NM_008566	Mcm5	3.38	0.0155	3422	chr8	+	77633426	77652338
NM_001081099	2610002D18Rik	3.34	0.0472	1270	chr4	+	134066913	134079837
NM_153404	Liph	3.33	0.0132	2050	chr16	-	21955463	21995523
NM_016777	Nasp	3.31	0.0439	3087	chr4	-	116273656	116300556
NM_009523	Wnt4	3.28	0.0402	1101	chr4	+	136833549	136852694
NM_009828	Ccna2	3.21	0.0295	2827	chr3	-	36463786	36470918
NM_011851	Nt5e	3.16	0.0479	3580	chr9	+	88222446	88266927
NM_001199955	Ankrd60	3.16	0.0013	598	chr2	-	173397890	173403842
NM_001039155	Triobp	3.14	0.0171	3807	chr15	+	78778153	78799272
NM_001033149	Ttc9	3.12	0.0162	2042	chr12	+	82732355	82765928
NM_010043	Des	3.10	0.0408	2162	chr1	+	75356918	75364290
NM_001195298	Kifc1	3.05	0.0010	2330	chr17	-	34012610	34027578
NM_146298	Olfr746	3.01	0.0162	945	chr14	+	51272913	51273858
NM_001085524	Gm5634	3.01	0.0489	842	chrX	-	8539259	8540101
NM_027290	Mcm10	2.96	0.0377	3510	chr2	-	4911769	4933837
NM_027411	Ccdc99	2.96	0.0203	2526	chr11	-	34622686	34647143
NM_009922	Cnn1	2.94	0.0276	1987	chr9	+	21903696	21913665
NM_024245	Kif23	2.94	0.0296	3439	chr9	-	61765084	61794606
NM_008563	Mcm3	2.93	0.0098	2886	chr1	-	20793094	20810294
NM_009104	Rrm2	2.85	0.0441	2199	chr12	+	25393118	25399011
NM_010615	Kif11	2.85	0.0315	4850	chr19	+	37450892	37496349
NM_172453	Pif1	2.80	0.0134	3680	chr9	+	65435011	65443769
NM_053173	Kifc5b	2.79	0.0009	2627	chr17	+	27054035	27069524
NM_016958	Krt14	2.78	0.0286	1680	chr11	-	100064475	100068824
NM_008652	Mybl2	2.76	0.0325	3702	chr2	+	162880370	162910423
NM_029835	5730590G19Rik	2.75	0.0445	7213	chr7	+	86805081	86843031
NM_001164253	Tpm1	2.74	0.0405	1675	chr9	-	66870399	66891678
NM_026785	Ube2c	2.72	0.0311	931	chr2	+	164595428	164598402
NM_029937	Nup210l	2.71	0.0025	5681	chr3	+	89908053	90015939
NM_022724	Suv39h2	2.71	0.0439	4282	chr2	-	3373086	3392258
NM_145150	Prc1	2.69	0.0315	3035	chr7	+	87439350	87461145
NM_009132	Scin	2.68	0.0012	2708	chr12	-	40786357	40860815
NM_009575	Zic3	2.65	0.0046	4035	chrX	+	55283804	55289807
NM_008567	Mcm6	2.64	0.0106	2901	chr1	-	130228167	130256233
NM_027182	Trip13	2.64	0.0410	2267	chr13	-	74049909	74075215

NM_011196	Ptger3	2.64	0.0281	2090	chr3	+	157229855	157307722
NM_012012	Exo1	2.64	0.0384	5506	chr1	+	177810908	177841527
NM_011234	Rad51	2.61	0.0486	2138	chr2	+	118938552	118961806
NM_001014976	Espl1	2.61	0.0074	6630	chr15	+	102126723	102154787
NM_009971	Csf3	2.59	0.0249	1363	chr11	+	98562626	98564943
NM_138673	Stab2	2.59	0.0065	8152	chr10	-	86303954	86470687
NM_001009940	Il19	2.57	0.0344	1066	chr1	-	132829232	132835818
NM_011623	Top2a	2.57	0.0184	5217	chr11	-	98854260	98885503
NM_009860	Cdc25c	2.57	0.0424	1941	chr18	-	34892650	34911187
NM_029662	Mfsd2a	2.56	0.0469	2166	chr4	-	122624093	122638431
NM_009863	Cdc7	2.55	0.0110	2960	chr5	+	107393340	107413450
NM_001037751	Defb48	2.55	0.0284	445	chr14	-	63596360	63603347
NM_197959	Kif18b	2.54	0.0341	3345	chr11	-	102766832	102786438
NM_001033484	Iqgap3	2.53	0.0382	5676	chr3	+	87885972	87924970
NM_001141977	Tpx2	2.53	0.0250	4365	chr2	+	152673699	152721057
NM_026560	Cdca8	2.52	0.0254	1626	chr4	-	124595708	124614161
NM_011634	Traip	2.52	0.0075	2697	chr9	+	107853293	107874599
NM_007681	Cenpa	2.52	0.0324	1337	chr5	+	30969274	30977199
NM_011497	Aurka	2.52	0.0286	1905	chr2	-	172181695	172196006
NM_023058	Pkmyt1	2.50	0.0038	2039	chr17	+	23863302	23873696
NM_007634	Ccnf	2.50	0.0276	3099	chr17	-	24360176	24388354
NM_144553	Dlgap5	2.50	0.0430	2966	chr14	-	48007453	48038082
NM_011015	Orc1	2.49	0.0500	3027	chr4	+	108252058	108287436
NM_028232	Sgol1	2.48	0.0488	3609	chr17	-	53814111	53828640
NM_030712	Cxcr6	2.48	0.0270	1893	chr9	+	123715594	123720872
NM_008506	Mycl1	2.47	0.0164	3518	chr4	+	122673341	122679723
NM_026410	Cdca5	2.47	0.0213	1906	chr19	+	6085096	6091773
NM_027650	Speer3	2.46	0.0311	1191	chr5	+	13791618	13796819
NM_001160262	Fam78b	2.45	0.0127	4986	chr1	+	168931547	169021433
NM_172578	Mis18bp1	2.44	0.0472	4038	chr12	-	66233720	66273567
NM_011131	Pold1	2.44	0.0073	3428	chr7	-	51788113	51804185
NM_008564	Mcm2	2.42	0.0138	3381	chr6	-	88833467	88848774
NM_011495	Plk4	2.42	0.0146	3567	chr3	+	40603872	40620805
NM_007526	Barx1	2.41	0.0172	1366	chr13	+	48758404	48761876
NM_013787	Skp2	2.40	0.0371	3204	chr15	-	9041741	9070207
NM_020034	Hist1h1b	2.39	0.0053	672	chr13	-	21871751	21872423
NM_009022	Aldh1a2	2.38	0.0329	2264	chr9	+	71063595	71144050
NM_001040435	Tacc3	2.38	0.0365	2637	chr5	+	34000795	34014846
NM_153544	BC030867	2.38	0.0347	2543	chr11	+	102110195	102126497
NM_001113204	Ncam1	2.35	0.0271	6993	chr9	-	49310250	49607174
NM_008446	Kif4	2.34	0.0326	4712	chrX	+	97821403	97922610
NM_001146081	Fancb	2.34	0.0364	2984	chrX	+	161418523	161435204
NM_001081363	Cenpf	2.34	0.0332	11130	chr1	-	191464492	191511965
NM_024184	Asf1b	2.34	0.0397	1599	chr8	+	86479592	86494094
NM_013882	Gtse1	2.34	0.0065	2707	chr15	+	85690375	85707003
NM_172786	Il20ra	2.34	0.0247	2088	chr10	+	19432392	19479859
NM_001164250	Tpm1	2.33	0.0241	1702	chr9	-	66870399	66897020
NM_029898	Ankrd55	2.33	0.0296	2781	chr13	+	113078658	113174210
NM_001081306	Ptprz1	2.32	0.0393	8068	chr6	+	22825501	23002916
NM_008815	Etv4	2.32	0.0224	2336	chr11	-	101631055	101646624
NM_013538	Cdca3	2.30	0.0490	1488	chr6	+	124780193	124783719
NM_178309	Brip1	2.30	0.0307	6933	chr11	-	85871637	86014695
NM_026778	Cthrc1	2.29	0.0433	1164	chr15	+	38908477	38918665
NM_008565	Mcm4	2.29	0.0312	3589	chr16	-	15623989	15637493

NM_013749	Tnfrsf12a	2.28	0.0437	980	chr17	-	23812411	23814416
NM_008632	Mtap2	2.27	0.0407	5445	chr1	+	66221902	66489157
NM_017407	Spag5	2.25	0.0426	3822	chr11	+	78115092	78135956
NM_029368	1700029F09Rik	2.24	0.0241	1265	chr1	-	44143461	44159233
NM_025995	Fbxo5	2.24	0.0408	1820	chr10	+	4541075	4547383
NM_028109	Tpx2	2.24	0.0434	4352	chr2	+	152673699	152721057
NM_172616	C330027C09Rik	2.23	0.0475	3974	chr16	+	48994300	49019818
NM_028131	Cenpn	2.21	0.0235	1768	chr8	+	119445639	119465403
NM_030609	Hist1h1a	2.19	0.0438	747	chr13	+	23855536	23856283
NM_010006	Cyp2d9	2.19	0.0070	1651	chr15	+	82282806	82287257
NM_009928	Col15a1	2.18	0.0463	5335	chr4	+	47220883	47326037
NM_021288	Tyms	2.18	0.0300	3798	chr5	-	30384739	30400165
NM_001103182	Lin9	2.17	0.0098	2996	chr1	+	182571464	182620818
NM_001164485	Fam170b	2.17	0.0251	1694	chr14	+	33647147	33649974
NM_001029856	Atad5	2.16	0.0129	7272	chr11	+	79902901	79949293
NM_009171	Shmt1	2.16	0.0262	2074	chr11	-	60602398	60624767
NM_001162506	Troap	2.16	0.0357	2255	chr15	+	98905403	98913840
NM_027699	1700108M19Rik	2.16	0.0207	660	chr12	-	36934931	36944789
NM_029150	Spata16	2.16	0.0093	2011	chr3	+	26536552	26882134
NM_001039934	Mtap2	2.16	0.0218	5535	chr1	+	66221902	66489157
NM_001159513	H2afy	2.14	0.0101	1975	chr13	-	56174982	56236911
NM_001033170	Fam83e	2.13	0.0209	2900	chr7	+	52976589	52984862
NM_001034867	Pm20d2	2.13	0.0035	5578	chr4	-	33257381	33276712
NM_028083	Chaf1b	2.12	0.0237	1913	chr16	+	93884145	93906351
NM_010305	Gnai1	2.12	0.0427	3193	chr5	-	17770952	17866231
NM_026340	Pop1	2.12	0.0042	3303	chr15	+	34425065	34460408
NM_001080129	Tmpo	2.12	0.0212	3533	chr10	-	90610315	90634327
NM_001159317	Il1rap	2.11	0.0192	4031	chr16	+	26581790	26725233
NM_178184	Hist1h2an	2.11	0.0040	393	chr13	-	21878694	21879087
NM_011711	Fmnl3	2.11	0.0034	4428	chr15	-	99147653	99200897
NM_026024	Ube2t	2.10	0.0438	1079	chr1	+	136859153	136870714
NM_145142	Chst10	2.09	0.0063	3135	chr1	-	38920717	38955005
NM_177372	Dna2	2.09	0.0459	4122	chr10	+	62409776	62436936
NM_027435	Atad2	2.09	0.0231	5683	chr15	-	57925601	57966637
NM_013733	Chaf1a	2.08	0.0344	3308	chr17	+	56179838	56207449
NM_177357	Kalrn	2.08	0.0236	15439	chr16	-	33969158	34514113
NM_001080995	4632434I11Rik	2.08	0.0243	3450	chr7	-	100006036	100022742
NM_011284	Rpa2	2.07	0.0160	1782	chr4	+	132324274	132334661
NM_001033166	2700094K13Rik	2.07	0.0140	697	chr2	-	84509377	84510865
NM_134010	Nup107	2.07	0.0269	3092	chr10	-	117187698	117229761
NM_001081390	Palld	2.06	0.0368	3566	chr8	-	63993819	64381487
NM_001080130	Tmpo	2.05	0.0174	3413	chr10	-	90610315	90634327
NM_133815	Lbr	2.05	0.0043	3568	chr1	-	183745445	183772532
NM_181589	Ckap2l	2.04	0.0353	3154	chr2	-	129093945	129122948
NM_178211	Hist1h4k	2.04	0.0148	312	chr13	-	21842062	21842374
NM_010158	Khdrbs3	2.04	0.0253	1954	chr15	+	68758849	68923948
NM_013455	Acr	2.04	0.0462	1503	chr15	+	89398756	89405015
NM_178591	Nrg1	2.02	0.0230	2103	chr8	-	32928499	33028675
NM_007525	Bard1	2.02	0.0251	5448	chr1	-	71074108	71149546
NM_020567	Gmnn	2.01	0.0473	995	chr13	-	24843713	24853806
NM_016692	Incenp	2.01	0.0199	3217	chr19	-	9946786	9974023
NM_134184	Vmn1r16	2.01	0.0411	912	chr6	-	57272717	57273629
NM_198605	F630043A04Rik	2.01	0.0168	2260	chr14	-	58425397	58445000
NM_001037719	Btla	2.01	0.0140	3235	chr16	+	45224449	45253008

NM_009272	Srm	2.00	0.0213	1328	chr4	+	147965621	147968728
NM_001080711	Dfnb59	2.00	0.0085	1219	chr2	+	76488329	76496613
NM_001076789	Cbx5	2.00	0.0358	8801	chr15	-	103021976	103070247
