# Supplementary material for: Fibrogenic Activity of MECP2 Is Regulated by Phosphorylation in Hepatic Stellate Cells
Source: Gastroenterology. 2019 Nov;157(5):1398–1412.e9. doi: 10.1053/j.gastro.2019.07.029 (PMC6853276; doi:10.1053/j.gastro.2019.07.029)
Supplement: Supplementary Table 3 [file mmc3.pdf]

Supplementary Table 3. Differentially expressed (DE) Upregulated lncRNAs

Sequence name	FC	p-val	length	S	Ch	Start	End	Source*	Associated Gene
AK042398	10.96	0.038	1455	-	chr13	13681874	13683327	fantom3	NM_010748
AK042700	6.75	0.041	2340	-	chr4	42172661	42175000	fantom3	
AK039830	6.39	0.003	3332	+	chr3	15128606	15131937	NRED	
uc.183-	5.49	0.007	236	+	chr11	32580147	32580383	UCR	NM_134015
AK145320	5.15	0.020	2227	+	chr16	81871754	81873980	fantom3	
uc.289+	4.96	0.001	254	+	chr14	23564681	23564935	UCR	NM_028275
AK143356	4.75	0.017	2289	+	chr5	26408711	26410997	fantom3	NM_001177579
ENSMUST00000170876	4.73	0.028	1004	-	chr11	96607345	96608724	Ensembl	NM_001177899
ENSMUST00000170876	4.73	0.028	1004	-	chr11	96607345	96608724	Ensembl	NM_001177898
ENSMUST00000170876	4.73	0.028	1004	-	chr11	96607345	96608724	Ensembl	NM_001033186
ENSMUST00000167770	4.36	0.024	966	+	chr8	35157601	35159203	Ensembl	NM_011722
NR_001584	4.27	0.046	1121	+	chr5	14945293	14978541	RefSeq_NR	
ENSMUST00000169039	4.21	0.009	3539	+	chr4	42566009	42600570	Ensembl	
uc007bdt.1	4.16	0.017	487	+	chr1	59383514	59409292	UCSC_kg	NM_001033373
AK087836	4.15	0.023	1241	+	chr5	26341388	26342629	fantom3	
AK087893	3.88	0.027	1799	+	chr5	26423108	26424904	fantom3	
AK038167	3.88	0.004	2755	+	chr1	110313653	110316409	fantom3	
AK033305	3.82	0.043	1866	+	chr5	26340763	26342629	fantom3	
AK048921	3.78	0.048	1515	-	chr8	115092137	115093651	fantom3	NM_130457
ENSMUST00000121909	3.76	0.031	1569	+	chrX	111135605	111137174	Ensembl	
AK132740	3.69	0.037	1579	+	chr5	121835530	121837110	fantom3	NM_172275
AK132740	3.69	0.037	1579	+	chr5	121835530	121837110	fantom3	NM_001163470
ENSMUST00000157493	3.67	0.009	312	+	chr6	8389352	8389664	Ensembl	
NR_003564	3.64	0.001	812	-	chr11	88825980	88828231	RefSeq_NR	
AK050947	3.57	0.008	1870	-	chr12	8298631	8300500	fantom3	
AK045912	3.49	0.004	1413	-	chr3	24737848	24739261	fantom3	
AK134457	3.37	0.017	3169	+	chr5	26418740	26421912	fantom3	
uc008wjx.1	3.30	0.047	454	-	chr5	8092259	8116659	UCSC_kg	NM_001007221
uc008wjx.1	3.30	0.047	454	-	chr5	8092259	8116659	UCSC_kg	NM_001098225
uc008wjx.1	3.30	0.047	454	-	chr5	8092259	8116659	UCSC_kg	NM_001007220
NR_033506	3.26	0.024	3558	+	chr4	41763293	42104673	RefSeq_NR	NM_001199960
NR_033506	3.26	0.024	3558	+	chr4	41763293	42104673	RefSeq_NR	NM_001199963
NR_033506	3.26	0.024	3558	+	chr4	41763293	42104673	RefSeq_NR	NM_001199964
NR_033506	3.26	0.024	3558	+	chr4	41763293	42104673	RefSeq_NR	NM_001199966
NR_033506	3.26	0.024	3558	+	chr4	41763293	42104673	RefSeq_NR	NM_001177580
NR_033506	3.26	0.024	3558	+	chr4	41763293	42104673	RefSeq_NR	NM_001099348
NR_033506	3.26	0.024	3558	+	chr4	41763293	42104673	RefSeq_NR	NM_023052
NR_033506	3.26	0.024	3558	+	chr4	41763293	42104673	RefSeq_NR	NM_023052
NR_033506	3.26	0.024	3558	+	chr4	41763293	42104673	RefSeq_NR	NM_011335
NR_033506	3.26	0.024	3558	+	chr4	41763293	42104673	RefSeq_NR	NM_011335
NR_033506	3.26	0.024	3558	+	chr4	41763293	42104673	RefSeq_NR	NM_001193666
NR_033506	3.26	0.024	3558	+	chr4	41763293	42104673	RefSeq_NR	NM_001193666
NR_033506	3.26	0.024	3558	+	chr4	41763293	42104673	RefSeq_NR	NM_010550
NR_033506	3.26	0.024	3558	+	chr4	41763293	42104673	RefSeq_NR	NM_001199962
NR_033506	3.26	0.024	3558	+	chr4	41763293	42104673	RefSeq_NR	NM_001164046
NR_033506	3.26	0.024	3558	+	chr4	41763293	42104673	RefSeq_NR	NM_001199961
NR_033506	3.26	0.024	3558	+	chr4	41763293	42104673	RefSeq_NR	NM_001100596
NR_033506	3.26	0.024	3558	+	chr4	41763293	42104673	RefSeq_NR	NM_001193668
NR_033506	3.26	0.024	3558	+	chr4	41763293	42104673	RefSeq_NR	NM_001193668
NR_033506	3.26	0.024	3558	+	chr4	41763293	42104673	RefSeq_NR	NM_001085530
NR_033506	3.26	0.024	3558	+	chr4	41763293	42104673	RefSeq_NR	NM_001199959
NR_033506	3.26	0.024	3558	+	chr4	41763293	42104673	RefSeq_NR	NM_001199965
ENSMUST00000121026	3.22	0.005	360	+	chrX	138495397	138495757	Ensembl	
AK046587	3.19	0.029	1571	-	chr7	113033809	113035380	fantom3	
AK132557	3.18	0.015	3835	+	chr14	64456259	64460091	fantom3	

ENSMUST00000119221	3.11	0.032	343	-	chr4	126201573	126201916	Ensembl	NM_153177
ENSMUST00000131638	3.10	0.045	1216	+	chr18	60380496	60381712	Ensembl	NM_001033767
uc007qle.1	3.04	0.010	2940	-	chr13	50772139	50775593	UCSC_kg	
AK082796	2.99	0.017	1483	+	chr11	66849005	66850488	fantom3	
uc009rmk.1	2.96	0.023	439	+	chr9	107500683	107501122	UCSC_kg	NM_001042779
uc009rmk.1	2.96	0.023	439	+	chr9	107500683	107501122	UCSC_kg	NM_009153
ENSMUST00000101090	2.91	0.044	478	+	chr5	77357392	77357870	Ensembl	NM_172146
NR_033123	2.91	0.022	4551	-	chr4	41902018	41925853	RefSeq_NR	
AK140313	2.87	0.032	4953	-	chr15	68152942	68157895	fantom3	
MM9LINCRNAEXON11523-	2.85	0.002	444	-	chr13	28510817	28511261	lincRNA	
ENSMUST00000130159	2.84	0.001	458	-	chrX	76511897	76540620	Ensembl	
uc007aev.1	2.84	0.012	2257	-	chr1	3638391	3648985	UCSC_kg	NM_001011874
uc008ysj.1	2.81	0.045	669	+	chr5	112635825	112639018	UCSC_kg	
NR_003248	2.76	0.005	3732	-	chr9	98849573	98855729	RefSeq_NR	NM_012020
uc009iks.1	2.74	0.037	1133	-	chr7	105830845	105851438	UCSC_kg	NM_028410
ENSMUST00000172223	2.74	0.039	621	-	chr3	96205072	96205937	Ensembl	
ENSMUST00000155944	2.72	0.022	3543	-	chr4	42418154	42452715	Ensembl	
AK046186	2.72	0.003	1540	-	chr7	130618901	130620441	fantom3	
ENSMUST00000144604	2.72	0.012	741	-	chr4	101019135	101028853	Ensembl	
MM9LINCRNAEXON11522-	2.72	0.015	238	-	chr13	28510437	28510675	lincRNA	
MM9LINCRNAEXON10419-	2.71	0.034	394	-	chr6	31149982	31150376	lincRNA	
uc008skp.1	2.70	0.024	371	+	chr4	41763413	41763784	UCSC_kg	
ENSMUST00000171819	2.67	0.026	3539	+	chr4	41925874	41960431	Ensembl	
ENSMUST00000140782	2.66	0.003	1853	+	chr3	152329876	152344396	Ensembl	NM_001081277
AK134233	2.65	0.019	2190	-	chr8	122083619	122085806	fantom3	
MM9LINCRNAEXON10944-	2.64	0.037	1342	-	chr2	71581306	71582648	lincRNA	
uc008wth.1	2.62	0.046	777	-	chr5	26786609	26819829	UCSC_kg	
AK047207	2.62	0.039	526	+	chr4	41760540	41761066	fantom3	
uc008zxj.1	2.62	0.019	576	-	chr5	135487992	135489007	UCSC_kg	NM_145215
uc008zxj.1	2.62	0.019	576	-	chr5	135487992	135489007	UCSC_kg	NM_001190437
ENSMUST00000130639	2.62	0.027	909	-	chr17	8486230	8503917	Ensembl	NM_134114
ENSMUST00000130639	2.62	0.027	909	-	chr17	8486230	8503917	Ensembl	NM_018819
AK134642	2.60	0.029	1838	-	chr9_random	147587	149423	fantom3	
ENSMUST00000167606	2.59	0.028	2215	-	chr9	20412209	20416929	Ensembl	
ENSMUST00000115902	2.56	0.026	1386	-	chrUn_random	554393	555791	Ensembl	
uc009fxb.1	2.54	0.003	283	+	chr7	28554249	28555744	UCSC_kg	
ENSMUST00000141440	2.53	0.037	269	+	chr6	145004908	145008412	Ensembl	NM_007532
ENSMUST00000119405	2.51	0.045	1849	+	chr4	41764013	41765862	Ensembl	
ENSMUST00000119510	2.50	0.016	849	+	chr1	89921785	89922634	Ensembl	
ENSMUST00000115901	2.50	0.036	672	+	chr4	42106046	42106718	Ensembl	
ENSMUST00000146404	2.50	0.001	1675	+	chr2	38279521	38311371	Ensembl	
ENSMUST00000166295	2.49	0.025	1446	+	chrUn_random	610376	616170	Ensembl	
ENSMUST00000152279	2.47	0.021	1157	-	chr14	62238317	62301162	Ensembl	
ENSMUST00000099292	2.44	0.048	1743	-	chr2	143998885	144015026	Ensembl	
ENSMUST00000133960	2.43	0.017	2953	+	chr4	3173151	3185239	Ensembl	
ENSMUST00000117125	2.43	0.046	1284	-	chr4	41896151	41897435	Ensembl	
NR_026688	2.42	0.021	519	-	chr5	135487991	135489027	RefSeq_NR	NM_145215
NR_026688	2.42	0.021	519	-	chr5	135487991	135489027	RefSeq_NR	NM_001190437
ENSMUST00000053717	2.42	0.013	684	-	chr19	20660563	20661247	Ensembl	
MM9LINCRNAEXON12072-	2.42	0.035	2230	-	chr1	137562833	137565063	lincRNA	

AK052921	2.42	0.031	1526	+	chr6	66222498	66234839	NRED	
ENSMUST00000120408	2.41	0.036	822	-	chr2	177715238	177717405	Ensembl	
ENSMUST00000151294	2.39	0.015	731	-	chr7	111682121	111683365	Ensembl	
NR_015566	2.38	0.045	5195	+	chr1	196843592	196864102	RefSeq_NR	
ENSMUST00000144933	2.37	0.043	557	+	chr4	3174664	3175674	Ensembl	
AK158457	2.37	0.018	2833	-	chr3	96047474	96050303	fantom3	NM_178212
AK158457	2.37	0.018	2833	-	chr3	96047474	96050303	fantom3	NM_013549
AK158457	2.37	0.018	2833	-	chr3	96047474	96050303	fantom3	NM_178216
uc008ski.1	2.37	0.029	426	-	chr4	41758222	41758648	UCSC_kg	
AK142834	2.35	0.042	2388	+	chrX	118021841	118024227	fantom3	
CR515238	2.35	0.034	215	-	chr12	25852471	25852681	lincRNA	
uc008tsr.1	2.35	0.046	864	+	chr4	94742359	94822969	UCSC_kg	
MM9LINCRNAEXON11365+	2.34	0.046	324	+	chr15	85470594	85470918	lincRNA	
ENSMUST00000121568	2.34	0.024	512	-	chrX	87989884	87990401	Ensembl	
uc008wsy.1	2.33	0.006	2609	+	chr5	25005758	25008367	UCSC_kg	
AK145626	2.33	0.028	1756	-	chr4	42101119	42102873	fantom3	
AK086591	2.31	0.016	1002	+	chr1	92177032	92180487	fantom3	
ENSMUST00000151170	2.31	0.010	1607	+	chr6	17148106	17155693	Ensembl	
ENSMUST00000128654	2.30	0.014	1870	-	chr11	18745853	18772158	Ensembl	
MM9LINCRNAEXON12061+	2.29	0.008	70	+	chr1	122987123	122987193	lincRNA	
ENSMUST00000171522	2.28	0.036	3220	-	chr6	21935907	22017687	Ensembl	NM_001081351
ENSMUST00000151501	2.28	0.014	3326	+	chr14	70132590	70145676	Ensembl	NM_134078
ENSMUST00000121579	2.28	0.023	328	+	chr4	25346495	25346823	Ensembl	
uc007wgm.1	2.28	0.038	269	-	chr15	74826881	74828331	UCSC_kg	NM_010738
AK076168	2.24	0.004	2312	+	chr9	41427074	41429386	fantom3	
uc009qvo.1	2.23	0.049	1482	+	chr9	81525964	81538763	UCSC_kg	NM_010482
ENSMUST00000165103	2.22	0.036	4231	-	chr13	39037411	39043182	Ensembl	NM_134060
ENSMUST00000165103	2.22	0.036	4231	-	chr13	39037411	39043182	Ensembl	NM_001170431
ENSMUST00000165103	2.22	0.036	4231	-	chr13	39037411	39043182	Ensembl	NM_001170430
ENSMUST00000122214	2.22	0.007	226	+	chr4	122132452	122132678	Ensembl	
ENSMUST00000142634	2.21	0.010	608	-	chr4	100195851	100200406	Ensembl	NM_001033773
ENSMUST00000138007	2.21	0.033	3220	+	chr11	88579958	88583914	Ensembl	NM_054043
ENSMUST00000138007	2.21	0.033	3220	+	chr11	88579958	88583914	Ensembl	NM_001201341
ENSMUST00000130892	2.20	0.047	1250	-	chr5	110596526	110605523	Ensembl	NM_008146
ENSMUST00000130892	2.20	0.047	1250	-	chr5	110596526	110605523	Ensembl	NM_172717
AK043175	2.20	0.006	2258	-	chr4	98858830	98861087	fantom3	NM_175029
AK043175	2.20	0.006	2258	-	chr4	98858830	98861087	fantom3	NM_001145967
NR_028122	2.20	0.022	459	+	chr2	163298280	163298739	RefSeq_NR	NM_173397
AK085737	2.19	0.012	2426	-	chr17	74226578	74229003	fantom3	
ENSMUST00000154798	2.19	0.032	1045	-	chr11	102481109	102485730	Ensembl	
ENSMUST00000137057	2.19	0.012	656	-	chr6	129182643	129188000	Ensembl	
AK139146	2.16	0.002	964	-	chr11	3227122	3228084	fantom3	
ENSMUST00000117226	2.14	0.008	497	+	chr14	54349178	54349856	Ensembl	
MM9LINCRNAEXON10607-	2.14	0.032	2164	-	chr5	113627537	113629701	lincRNA	
AK083522	2.14	0.026	1019	+	chr2	167458749	167459769	fantom3	
ENSMUST00000119869	2.14	0.044	1048	+	chr4	84343766	84344814	Ensembl	
uc007qkb.1	2.14	0.025	2940	+	chr13	50357183	50360637	UCSC_kg	
ENSMUST00000132326	2.13	0.019	460	-	chr2	74541304	74550111	Ensembl	NM_010468
ENSMUST00000132326	2.13	0.019	460	-	chr2	74541304	74550111	Ensembl	NM_008276
AK039686	2.13	0.013	2347	-	chr6	128137835	128150641	NRED	
uc007bdq.1	2.13	0.040	395	+	chr1	59344613	59367703	UCSC_kg	NM_001033373
ENSMUST00000145799	2.12	0.001	511	-	chr2	74548249	74554053	Ensembl	NM_010468
ENSMUST00000118930	2.12	0.022	291	-	chr2	176386914	176387205	Ensembl	NM_001177543
ENSMUST00000163635	2.12	0.044	445	-	chr14	8597331	8606583	Ensembl	
AK033843	2.11	0.020	1237	+	chr7	19449514	19450751	fantom3	
uc008gfj.1	2.11	0.032	4277	-	chr19	5798394	5802671	UCSC_kg	
AK079652	2.10	0.026	1049	+	chr6	125196441	125197489	fantom3	

AK080602	2.10	0.006	2773	+	chr14	64471552	64474322	fantom3	
AK020198	2.10	0.017	1035	-	chr2	11259992	11320127	fantom3	
AK083796	2.10	0.050	2842	+	chr7	28565647	28568488	NRED	
ENSMUST00000139288	2.10	0.029	410	+	chr2	33033421	33037401	Ensembl	NM_175211
uc007mnh.1	2.09	0.035	626	+	chr11	117472658	117474111	UCSC_kg	
uc008smn.1	2.09	0.027	684	+	chr4	42415893	42416577	UCSC_kg	
BY031985	2.08	0.033	470	-	chr14	92394267	92394664	lincRNA	
ENSMUST00000127631	2.07	0.009	942	+	chr8	70480668	70501950	Ensembl	NM_177698
AK053656	2.07	0.002	4598	-	chr8	118211623	118216217	fantom3	
ENSMUST00000118430	2.06	0.012	226	+	chr4	121870821	121871047	Ensembl	
MM9LINCRNAEXON104<br>85-	2.06	0.022	1649	-	chr6	99561279	99562928	lincRNA	
AK043941	2.06	0.032	924	-	chr7	111411900	111412824	fantom3	
uc007iyy.1	2.06	0.013	520	+	chr11	54950545	54953695	UCSC_kg	NM_172258
ENSMUST00000120530	2.06	0.012	342	-	chr17	92438999	92439341	Ensembl	
uc007zaj.1	2.06	0.028	3017	+	chr16	33755635	33768281	UCSC_kg	NM_175256
humanlincRNA2392-	2.05	0.027	9266	+	chrX	152944622	152953888	lincRNA	
ENSMUST00000123905	2.04	0.044	659	-	chr16	30969478	30973604	Ensembl	NM_198626
ENSMUST00000139653	2.04	0.017	675	+	chr4	141640770	141643992	Ensembl	NM_145402
AK043325	2.04	0.025	1003	-	chr1	190912396	190913399	fantom3	
ENSMUST00000161461	2.04	0.035	793	+	chr1	162965308	162968670	Ensembl	NM_173424
NR_015533	2.04	0.042	2006	-	chr7	68850735	69072460	RefSeq_NR	
AK081202	2.03	0.020	2082	-	chr1	196863022	196865102	fantom3	
AK082426	2.03	0.015	1754	+	chr17	70679696	70681450	fantom3	
MM9LINCRNAEXON105<br>07-	2.03	0.050	176	-	chr6	129188203	129188379	lincRNA	
uc009aux.1	2.03	0.010	2991	-	chr6	3283193	3296069	UCSC_kg	
MM9LINCRNAEXON112<br>80-	2.03	0.050	2861	-	chr16	30180887	30183748	lincRNA	
uc007gjn.1	2.02	0.018	490	+	chr10	81816999	81863591	UCSC_kg	
ENSMUST00000121541	2.02	0.032	279	+	chr8	22358662	22359442	Ensembl	
AK082404	2.02	0.042	1340	+	chr5	120876868	120878207	fantom3	
MM9LINCRNAEXON117<br>01-	2.01	0.023	1027	-	chr12	85220758	85221785	lincRNA	
AK016911	2.00	0.017	1450	-	chr10	75298720	75300169	fantom3	
AK017999	2.00	0.020	1357	+	chr6	125196422	125197490	fantom3	
ENSMUST00000120181	2.00	0.024	324	+	chr2	176526876	176527200	Ensembl	NM_001177543

The table contains the following information about the DE upregulated lncRNAs: Sequence name (the sequence identifier of the LncRNA), FC (Absolute Fold change is the absolute ratio (no log scale) of normalized intensities between two conditions), p-val (P-value, P-value calculated from t-test), length (RNA length), Chr (chromosome), S (DNA strand), Start and End (coding or non-coding genome location), , Source of the collection and Associated gene IS (the Accession number of the associated coding gene to the lncRNA).

* Source of LncRNA collection:
- RefSeq_NR: RefSeq validated non-coding RNA
- UCSC_kg: UCSC known genes annotated as "non-coding", "near-coding" and "antisense" (http://genome.ucsc.edu/cgi-bin/hgTables/)
- Ensembl: Ensembl (http://www.ensembl.org/index.html)
- Fantom3: Fantom project (http://fantom.gsc.riken.jp/)
- RNAdb: RNAdb2.0 (http://research.imb.uq.edu.au/rnadb/)
- NRED: NRED (http://jsm-research.imb.uq.edu.au/nred/cgi-bin/ncrnadb.pl)
- UCR: "ultra-conserved region" among human, mouse and rat (http://users.soe.ucsc.edu/~jill/ultra.html)
- lincRNA: lincRNA identified by John Rinn's group (Guttman et al. 2009; Khalil et al. 2009)
