# Supplementary material for: Fibrogenic Activity of MECP2 Is Regulated by Phosphorylation in Hepatic Stellate Cells
Source: Gastroenterology. 2019 Nov;157(5):1398–1412.e9. doi: 10.1053/j.gastro.2019.07.029 (PMC6853276; doi:10.1053/j.gastro.2019.07.029)
Supplement: Supplementary Table 4 [file mmc4.pdf]

Supplementary Table 4. Differentially expressed (DE) Downregulated lncRNAs

Sequence name	FC	p-val	length	S	Ch	Start	End	Source*	Associated gene ID
ENSMUST00000116395	9.65	0.0002	240	-	chrX	72117534	72117774	Ensembl	
uc009uoz.1	4.34	0.003	2940	-	chrX	147726516	147779389	UCSC_kg	NM_198105
humanlincRNA2046-	3.86	0.024	18943	+	chr13	17931594	17950537	lincRNA	
NR_028427	3.85	0.029	5292	-	chr19	53515701	53539286	RefSeq_NR	
AK039897	3.65	0.024	903	+	chr6	120901981	120902884	fantom3	
MM9LINCRNAEXON11597+	3.60	0.013	327	+	chr13	83869681	83870008	lincRNA	
humanlincRNA1512-	3.49	0.005	5490	+	chr5	37112733	37118223	lincRNA	
humanlincRNA1948+	3.49	0.011	26171	+	chr10	30142092	30168263	lincRNA	
MM9LINCRNAEXON11652-	3.41	0.012	802	-	chr12	12895177	12895979	lincRNA	
mouselincRNA0294-	3.30	0.035	15295	+	chr12	10663990	10679285	lincRNA	
MM9LINCRNAEXON11105+	3.23	0.016	493	+	chr19	27461455	27461948	lincRNA	
ENSMUST00000166710	3.22	0.034	614	+	chr5	13811241	13814452	Ensembl	
uc007pzn.1	3.22	0.034	2852	-	chr13	31670024	31717661	UCSC_kg	NM_010225
uc008hgf.1	3.19	0.014	1210	-	chr19	34314826	34316036	UCSC_kg	NM_007392
ENSMUST00000120731	3.17	0.034	1585	+	chr4	54865999	54867584	Ensembl	
BY307334	3.05	0.009	377	-	chr13	34873453	34906413	lincRNA	
MM9LINCRNAEXON10055-	3.03	0.050	345	-	chrX	135890738	135891083	lincRNA	
ENSMUST00000119494	3.02	0.036	1788	+	chr5	35896832	35898620	Ensembl	
ENSMUST00000121232	2.95	0.019	1527	-	chrX	8239427	8240954	Ensembl	
ENSMUST00000117514	2.91	0.028	1493	-	chr16	91513385	91514878	Ensembl	
ENSMUST00000140978	2.88	0.008	654	+	chr2	69876967	69880463	Ensembl	NM_177376
uc007zfw.1	2.76	0.018	4098	-	chr16	42725814	42875693	UCSC_kg	
uc007ocn.1	2.75	0.046	4628	+	chr12	82732355	82768538	UCSC_kg	NM_001033149
ENSMUST00000117166	2.68	0.010	1344	-	chr2	6053253	6055002	Ensembl	
uc008uju.1	2.67	0.025	6387	-	chr4	118035347	118056021	UCSC_kg	NM_026601
uc008uju.1	2.67	0.025	6387	-	chr4	118035347	118056021	UCSC_kg	NM_198170
AK136349	2.63	0.021	1102	-	chr10	68797903	68799006	fantom3	
MM9LINCRNAEXON11230-	2.62	0.002	885	-	chr17	66831155	66832040	lincRNA	
uc008sir.1	2.61	0.006	1112	-	chr4	41401422	41411881	UCSC_kg	NM_025539
uc008sir.1	2.61	0.006	1112	-	chr4	41401422	41411881	UCSC_kg	NM_024241
uc008uxa.1	2.61	0.030	1747	-	chr4	129118294	129124212	UCSC_kg	
ENSMUST00000121946	2.59	0.043	2902	-	chr6	38492302	38496074	Ensembl	
ENSMUST00000152745	2.59	0.050	1261	+	chr5	23356414	23360742	Ensembl	
AK135697	2.53	0.029	2877	+	chr10	112412677	112465278	fantom3	
mouselincRNA0154+	2.48	0.043	5300	+	chr10	24333365	24338665	lincRNA	
AK014021	2.48	0.040	1294	+	chr2	109858118	110013517	lincRNA	NM_026613
mouselincRNA1616+	2.46	0.017	9450	+	chrX	45398900	45408350	lincRNA	
uc008atc.1	2.43	0.009	270	+	chr17	23873321	23873696	UCSC_kg	NM_023058
uc008atc.1	2.43	0.009	270	+	chr17	23873321	23873696	UCSC_kg	NM_023824
ENSMUST00000166061	2.42	0.020	1153	-	chr4	145914739	145915894	Ensembl	
ENSMUST00000165496	2.42	0.038	289	+	chr12	108657350	108657639	Ensembl	
ENSMUST00000152660	2.42	0.016	300	+	chr8	71144036	71144336	Ensembl	NM_172753
MM9LINCRNAEXON11665+	2.41	0.013	1574	+	chr12	33824347	33825921	lincRNA	
uc007kde.1	2.41	0.029	863	+	chr11	74986061	74991135	UCSC_kg	NM_027136
ENSMUST00000165118	2.40	0.023	1153	-	chr4	146460094	146461249	Ensembl	NM_027285
uc009bpx.1	2.40	0.030	734	+	chr6	41493458	41498351	UCSC_kg	
AK131677	2.37	0.038	631	+	chr13	24853436	24854065	fantom3	NM_020567
AK090241	2.34	0.021	2526	-	chr16	25943095	25945619	fantom3	
MM9LINCRNAEXON10457-	2.34	0.046	197	-	chr6	67213651	67213848	lincRNA	
ENSMUST00000117910	2.33	0.048	477	-	chr2	4755181	4755658	Ensembl	
AK007126	2.33	0.042	413	+	chr12	92208388	92209958	fantom3	
ENSMUST00000084861	2.31	0.023	1091	+	chr4	145632856	145633949	Ensembl	
ENSMUST00000120764	2.27	0.017	505	+	chr8	102584015	102584520	Ensembl	
uc009gft.1	2.25	0.004	2105	-	chr7	31438730	31449988	UCSC_kg	NM_027999

MM9LINCRNAEXON10746-	2.25	0.022	1505	-	chr4	124288832	124290337	lincRNA	
ENSMUST00000166606	2.25	0.002	698	+	chr5	100406704	100407877	Ensembl	NM_007516
ENSMUST00000166606	2.25	0.002	698	+	chr5	100406704	100407877	Ensembl	NM_001077266
ENSMUST00000166606	2.25	0.002	698	+	chr5	100406704	100407877	Ensembl	NM_001077267
ENSMUST00000166606	2.25	0.002	698	+	chr5	100406704	100407877	Ensembl	NM_001077265
uc007qbc.1	2.24	0.038	1033	-	chr13	34176023	34205141	UCSC_kg	
ENSMUST00000058584	2.24	0.020	4116	+	chr15	60655895	60663348	Ensembl	NM_001162926
ENSMUST00000119302	2.21	0.041	1984	+	chr1	67631626	67633610	Ensembl	
NR_028404	2.20	0.004	4330	-	chrX	99261880	99266646	RefSeq_NR	NM_183318
NR_028404	2.20	0.004	4330	-	chrX	99261880	99266646	RefSeq_NR	NM_001163610
uc009kdq.1	2.19	0.030	1463	-	chr7	141376578	141423814	UCSC_kg	NM_007400
AK051578	2.18	0.017	3037	+	chr1	91826835	91829865	fantom3	NM_010262
AK020521	2.16	0.011	388	+	chr2	70550040	70550427	fantom3	
ENSMUST00000162565	2.16	0.047	867	+	chr2	93292976	93296878	Ensembl	NM_001136055
ENSMUST00000162565	2.16	0.047	867	+	chr2	93292976	93296878	Ensembl	NM_007656
uc008wvu.1	2.15	0.028	1482	+	chr5	30969274	30977199	UCSC_kg	NM_007681
humanlincRNA2338-	2.15	0.038	9701	+	chr4	39054681	39064382	lincRNA	
ENSMUST00000116301	2.14	0.006	411	+	chr11	58775313	58775724	Ensembl	
BX521937	2.14	0.035	530	+	chr1	35034878	35035099	lincRNA	
AK201973	2.14	0.031	221	+	chr1	91828299	91828518	NRED	NM_010262
AK142421	2.13	0.011	3373	-	chr4	144999190	145002564	fantom3	
ENSMUST00000141637	2.12	0.050	512	+	chr4	84387378	84388195	Ensembl	
ENSMUST00000121775	2.10	0.020	661	-	chr11	40236195	40236856	Ensembl	
uc.461+	2.10	0.010	397	-	chrX	90703238	90703635	UCR	NM_008892
AK076567	2.06	0.019	910	-	chr1	9657495	9658406	fantom3	
uc007gnh.1	2.05	0.050	483	+	chr10	85391518	85408951	UCSC_kg	NM_001163614
uc007gnh.1	2.05	0.050	483	+	chr10	85391518	85408951	UCSC_kg	NM_145422
AK033479	2.05	0.027	3562	-	chr1	146867202	146870764	fantom3	
AK041010	2.04	0.017	1086	+	chr4	32326403	32327486	predicted	
uc007dkt.1	2.04	0.018	4316	+	chr1	168931605	169021141	UCSC_kg	NM_001160262
uc007dkt.1	2.04	0.018	4316	+	chr1	168931605	169021141	UCSC_kg	NM_175461
uc007dkt.1	2.04	0.018	4316	+	chr1	168931605	169021141	UCSC_kg	NM_001160261
ENSMUST00000119909	2.03	0.017	387	-	chr2	165750765	165751341	Ensembl	
ENSMUST00000168749	2.03	0.035	1091	-	chr4_random	41109	42202	Ensembl	
MM9LINCRNAEXON11621+	2.02	0.039	2759	+	chr13	98280390	98283149	lincRNA	
ENSMUST00000119135	2.02	0.039	1371	+	chr7	48745720	48747091	Ensembl	NM_001033530
AW557181	2.02	0.005	487	+	chr1_random	31011	31498	lincRNA	
ENSMUST00000129696	2.01	0.025	639	+	chr1	38364746	38368775	Ensembl	NM_010678
AK143705	2.00	0.033	2611	+	chr10	96114479	96117090	fantom3	

The table contains the following information about the DE downregulated lncRNAs: Sequence name (the sequence identifier of the LncRNA), FC (Absolute Fold change is the absolute ratio (no log scale) of normalized intensities between two conditions), p-val (P-value, P-value calculated from t-test), length (RNA length), Chr (chromosome), S

(DNA strand), Start and End (coding or non-coding genome location), , Source of the collection and Associated gene IS (the Accession number of the associated coding gene to the lncRNA).

* Source of LncRNA collection:
- RefSeq_NR: RefSeq validated non-coding RNA
- UCSC_kg: UCSC known genes annotated as "non-coding", "near-coding" and "antisense" (http://genome.ucsc.edu/cgi-bin/hgTables/)
- lincRNA: lincRNA identified by John Rinn's group (Guttman et al. 2009; Khalil et al. 2009)

* Source of LncRNA collection:
- RefSeq_NR: RefSeq validated non-coding RNA
- UCSC_kg: UCSC known genes annotated as "non-coding", "near-coding" and "antisense" (http://genome.ucsc.edu/cgi-bin/hgTables/)
- Ensembl: Ensembl (http://www.ensembl.org/index.html)
- Fantom3: Fantom project (http://fantom.gsc.riken.jp/)
- RNAdb: RNAdb2.0 (http://research.imb.uq.edu.au/rnadb/)
- NRED: NRED (http://jsm-research.imb.uq.edu.au/nred/cgi-bin/ncrnadb.pl)
- UCR: "ultra-conserved region" among human, mouse and rat (http://users.soe.ucsc.edu/~jill/ultra.html)
- lincRNA: lincRNA identified by John Rinn's group (Guttman et al. 2009; Khalil et al. 2009)
