# Supplementary material for: Fibrogenic Activity of MECP2 Is Regulated by Phosphorylation in Hepatic Stellate Cells
Source: Gastroenterology. 2019 Nov;157(5):1398–1412.e9. doi: 10.1053/j.gastro.2019.07.029 (PMC6853276; doi:10.1053/j.gastro.2019.07.029)
Supplement: Supplementary Table 7 [file mmc7.pdf]

Supplementary Table 7. Quantitative PCR primers used for lncRNA corroboration and protein-coding RNAs validation.

Gene Symbol	Sequence Name	Forward and reverse primer pair sequences	Anneal temp	bp product
**AK080187**	uc008hgf.1	GAAGGGGGAAAACGGCAGTA	57	142
		TGCATTTTTGGGGACACCCT		
**Has2**	NM_008216	AGTCCCTTGAAACCCCGATT	57	161
		GTCGTCTGAGTTCCCATCGA		
**Myl7**	NM_022879	AGCTCGGGAGGGTAAGTGTT	57	112
		CCATTGAGCTTCTCCCCGAA		
**Des**	NM_010043	CAAGGGCTCCTCGAGTTCAA	57	174
		AACTCCTGGTTCACAGCGTC		
**Acan**	NM_007424	GAACTTGGCCATGGTCCTTC	57	161
		GGGTAAGCAGACAGGTCACT		
**Cdk15**	NM_001033373.2	CAGAAGCATCCAGTTCCACG	57	128
		CCCCTGCCTCAGATTCTCTTC		
**Tnxb**	NM_031176.2	CCAAGTCCTGTCCCGAAGAC	57	223
		CGTCCTCTCTGGTTGCAGTT		
**Sepp1**	NM_009155	TGACAGTGTGCGGAAAACCT	57	116
		GGCTGATTTTGTCAGGCAGC		
**Ostn**	NM_198112.2	GCCACTGAGCTTTCGGCTAA	57	120
		GAGTCTGTCAAGGGGAGACC		
**Has1**	NM_008215.2	TCTTTGCCCTGCTCATCCTG	57	122
		TAGGTGTGCGCTGAGGAATG		
**Has3**	NM_008217.4	GCTTCTTTGTGTGGCGTAGC	57	189
		AGTCCACTGAGTTGCCAAGG		
**Mcm6**	NM_008567	TGCTCACCCGAATCAGTGG	57	159
		CACACACTGGATTTCGGCAG		
**Mcm5**	NM_008566	CATCCGGAGCTCGTACATCC	57	285
		GGGTCTCCCAACATCAGCAA		
**Mcm4**	NM_008565	CCAATCGGAGACGTAGAGGC	57	281
		CACCTGCAAACCCTTTCGTG		
**Mcm3**	NM_008563	CGAGGAGGACCAAGGCATTT	57	120
		AGGCGGTTAGCCCTCTTTTC		
**Mcm2**	NM_008564	AAGTGTCTAGCCGTGCCATC	57	151
		TCGTCAATGAGACACACCCC		
**Eme1**	NM_177752	GCAGTAGCAGTGAGGACGAA	57	178
		ATCTTTGGCCATGGTTGCCT		
**Brip1**	NM_178309	CACTGTTTGCTGGAGAGTCC	57	188
		TCCAGGTTTGTGTCGCTGTAG		
**Rad51**	NM_011234	AGGGTTCAACACAGACCACC	57	153
		TGCATTTGCCTGGCTGAAAG		
**Ccna2**	NM_009828	TGTCACTGCTGGTCCTTCAT	57	157
		TCCGGGTAAAGAGACAGCTG		
**Pold1**	NM_011131	ATTCTTGCGTCTGGCACTCA	57	264
		TGGAAGGAGGAGTCACGGAT		

**Dna2**	NM_177372	GCGGAAAAATGGTCCCATGC	57	131
		AGGTAACGGCTGCCTTGTTC		
**Rpa2**	NM_011284	GAGTTCACCGCACACATCCT	57	154
		GGCCATTTGCTGGCATGAAG		
**Col1a1 (rat)**	NM_001145366.1	CTGACTGGAAGAGCGGAGAG	58	113
		CTGAGTGGGGAACACACAGG		
**TGFβ (rat)**	NM_021578.2	GACTCTCCACCTGCAAGACC	58	100
		GGACTGGCGAGCCTTAGTTT		
**TIMP-1 (rat)**	NM_053819.1	TCTGGCATCCTCTTGTTGCT	58	100
		TAACCAGGTCCGAGTTGCAG		
**MMP13**	NM_008607	TGTTTGCAGAGCACTACTTGAA	60	132
		CAGTCACCTCTAAGCCAAAGAAA		
**TNFα**	NM_013693	CCCTCACACTCAGATCATCTTCT	60	61
		GCTACGACGTGGGCTACAG		
**IL-1b**	NM_008361	CAACCAACAAGTGATATTCTCCATG	60	152
		GATCCACACTCTCCAGCTGCA		
**IL-6**	NM_031168	TAGTCCTTCCTACCCCAATTTCC	60	76
		TTGGTCCTTAGCCACTCCTTC		
**MIP2α/ CXCL2**	NM_009140	CCAACCACCAGGCTACAGG	60	108
		GCGTCACACTCAAGCTCTG		
**GAPDH**	NM_008084	GCACAGTCAAGGCCGAGAAT	57	151
		GCCTTCTCCATGGTGGTGAA		
**βactin**	NM_031144.3	AGCCATGTACGTAGCCATCC	57	228
		CTCTCAGCTGTGGTGGTGAA		
